# Unraveling Synaptic GCaMP Signals: Differential Excitability and Clearance Mechanisms Underlying Distinct Ca^2+^ Dynamics in Tonic and Phasic Excitatory, and Aminergic Modulatory Motor Terminals in *Drosophila*

**DOI:** 10.1523/ENEURO.0362-17.2018

**Published:** 2018-02-19

**Authors:** Xiaomin Xing, Chun-Fang Wu

**Affiliations:** Department of Biology, University of Iowa, Iowa City, IA 52242

**Keywords:** ion channels, mitochondria, octopamine, PMCA, residual calcium, synaptic plasticity

## Abstract

GCaMP is an optogenetic Ca^2+^ sensor widely used for monitoring neuronal activities but the precise physiological implications of GCaMP signals remain to be further delineated among functionally distinct synapses. The *Drosophila* neuromuscular junction (NMJ), a powerful genetic system for studying synaptic function and plasticity, consists of tonic and phasic glutamatergic and modulatory aminergic motor terminals of distinct properties. We report a first simultaneous imaging and electric recording study to directly contrast the frequency characteristics of GCaMP signals of the three synapses for physiological implications. Different GCaMP variants were applied in genetic and pharmacological perturbation experiments to examine the Ca^2+^ influx and clearance processes underlying the GCaMP signal. Distinct mutational and drug effects on GCaMP signals indicate differential roles of Na^+^ and K^+^ channels, encoded by genes including *paralytic* (*para*), *Shaker* (*Sh*), *Shab*, and *ether-a-go-go* (*eag*), in excitability control of different motor terminals. Moreover, the Ca^2+^ handling properties reflected by the characteristic frequency dependence of the synaptic GCaMP signals were determined to a large extent by differential capacity of mitochondria-powered Ca^2+^ clearance mechanisms. Simultaneous focal recordings of synaptic activities further revealed that GCaMPs were ineffective in tracking the rapid dynamics of Ca^2+^ influx that triggers transmitter release, especially during low-frequency activities, but more adequately reflected cytosolic residual Ca^2+^ accumulation, a major factor governing activity-dependent synaptic plasticity. These results highlight the vast range of GCaMP response patterns in functionally distinct synaptic types and provide relevant information for establishing basic guidelines for the physiological interpretations of presynaptic GCaMP signals from in situ imaging studies.

## Significance Statement

GCaMPs are a family of genetically encoded Ca^2+^ indicators widely employed in monitoring neuronal circuit activities. This study employed a genetic model system to enable simultaneous recording of presynaptic GCaMP signals in three functionally distinct types of synapses side by side. The results reveal how differential control by membrane excitability and mitochondria-powered Ca^2+^ clearance mechanisms shape distinct residual Ca^2+^ dynamics in different synaptic boutons during repetitive firing. Our results demonstrate a wide range of GCaMP response patterns in relation to different types of synaptic transmission and thereby provide background information for proper interpretations of GCaMP signals in a variety of synaptic activities.

## Introduction

Ca^2+^ influx on action potential arrival at synaptic terminals triggers neurotransmitter release, and residual Ca^2+^ accumulation following repetitive action potentials regulates activity-dependent synaptic plasticity (Katz and Miledi, 1967, 1968; Zucker and Regehr, 2002). Na^+^ and K^+^ channels play fundamental roles in shaping the axonal action potential and its repetitive firing pattern (Hille, 2001) and thus can profoundly influence the amplitudes and kinetics of synaptic Ca^2+^ elevation. Conversely, Ca^2+^ clearance mechanisms, including mitochondrial and endoplasmic reticulum (ER) sequestration (Tang and Zucker, 1997; Suzuki et al., 2002; Klose et al., 2009) and energy-dependent extrusion via plasma membrane Ca^2+^-ATPase (PMCA; Dipolo and Beaugé, 1979; Lnenicka et al., 2006), are critical in the restoration of synaptic basal Ca^2+^ levels.

GCaMPs are widely used genetically encoded Ca^2+^ indicators (Nakai et al., 2001; Chen et al., 2013). Despite the frequent applications of GCaMPs in monitoring neuronal activities in nervous systems of various animal species (Wang et al., 2003; Reiff et al., 2005; Rickgauer et al., 2014), it is unclear how differences in membrane excitability and Ca^2+^ clearance mechanisms determine the amplitude and kinetics of GCaMP Ca^2+^ signals in functionally distinct categories of synapses.

We analyzed GCaMP signals in the *Drosophila* larval neuromuscular junction (NMJ), in which both excitatory (glutamatergic tonic type Ib and phasic type Is) as well as modulatory (octopaminergic type II) synapses could be monitored simultaneously within the same optical microscopy field (Johansen et al., 1989; Keshishian et al., 1993; Kurdyak et al., 1994; Monastirioti et al., 1995; Hoang and Chiba, 2001). The glutamatergic type I synapses have been extensively studied for their electrophysiological properties (Jan and Jan, 1976; Ueda and Wu, 2006, 2012; Berke et al., 2013) and striking phenotypes caused by ion channel mutations (for review, see Ganetzky and Wu, 1986; Fox et al., 2005; Frolov et al., 2012). Octopaminergic type II synaptic terminals are known to modulate the growth and transmission properties of type I synapses (Koon et al., 2011) and to display remarkable excitability-dependent plasticity (Budnik et al., 1990; Zhong et al., 1992; Zhong and Wu, 2004). However, differences in excitability control and Ca^2+^ handling properties among these three distinct synaptic types remain to be determined.

This decade-long study, extended from earlier results (Ueda and Wu, 2006, 2009a; Xing, 2014), employed different versions of GCaMPs, including GCaMPs 1, 5, and 6, to delineate the distinct frequency characteristics of GCaMP signals from type Ib, Is, and II synapses and their preferential sensitivities to different pharmacological or genetic perturbations. In particular, our results show that type II synapses were most strongly affected by manipulations of channels encoded by *ether-a-go-go* (*eag*, Eag, or K_V_10 ortholog; for details, see Materials and Methods), *Shab* (K_V_2 ortholog), and *paralytic* (*para*, Na_V_1) channels, whereas type Is synapses were most severely modified by manipulations of *Shaker* (*Sh*, K_V_1 ortholog). Strikingly, double insults through manipulating *Sh* together with either *eag* or *Shab* could generate extreme hyperexcitability in type Is synapses, leading to greatly enhanced GCaMP signals on individual nerve stimulation. In contrast, type Ib synapses remained largely intact in the above experimentations but could display similar extreme hyperexcitability following triple insults with combinations of mutations or blockers of K^+^ channels. Simultaneous focal electrical recordings of synaptic activities revealed that such extreme cases of enhanced GCaMP signals actually resulted from supernumerary high-frequency (>100 Hz) repetitive firing in the motor terminals following each single stimulus.

Further kinetic analysis revealed different Ca^2+^ clearance capacity among three types of synaptic terminals. We found that Na^+^ and K^+^ channel mutations or blockers influence mainly the rise kinetics of GCaMP signals, whereas inhibiting Ca^2+^ clearance mediated by PMCA (via high pH treatment) slowed the decay phase acutely. In addition, we discovered that long-term inhibition of mitochondrial energy metabolism by incubation with either 2,4-dinitrophenol (DNP; cf. Greenawalt et al., 1964) or azide (cf. Bowler et al., 2006) led to drastically lengthened decay time of the GCaMP signal and significantly altered its frequency responses to repetitive stimulation, over a time course of tens of minutes.

Overall, this study demonstrates a wide range of GCaMP response patterns indicating differential membrane excitability and Ca^2+^ clearance mechanisms in functionally distinct types of synapses. Although the slow kinetics of GCaMP signals could not adequately resolve the rapid process of Ca^2+^ influx triggered by individual action potentials, they could nevertheless report cytosolic residual Ca^2+^ accumulation on repetitive synaptic activities. Our data thus provide essential baseline information for refined interpretations of GCaMP signals when monitoring *in vivo* neural circuit activities that often result from interplay among different categories of synapses.

## Materials and Methods

### Fly stocks

All stocks were maintained at room temperature (22–24°C). The Gal4-UAS system was employed for targeted expression of different *GCaMP* versions in the motor neurons. Homozygous stocks were first constructed to carry several combinations of the particular Gal4 driver and UAS-*GCaMP* responder. For instance, a fly strain carrying *UAS-GCaMP1.3* (a gift from Dr. Yalin Wang and Dr. Yi Zhong of Cold Spring Harbor Laboratory; cf. Wang et al., 2004; Ueda and Wu, 2006) was recombined with a motor-neuron expressing Gal4 driver P{GawB}*c164* (Torroja et al., 1999), forming a fly strain +; *c164Gal4-UAS-GCaMP1.3* (referred to as *c164-GCaMP1.3*). Similarly, *UAS-GCaMP6m* (Bloomington stock center; Chen et al., 2013) was recombined with *nsynaptobrevin-Gal4* (*nSyb-Gal4*, a gift from Dr. Toshihiro Kitamoto, University of Iowa), forming the strain +; +; *nSyb-GCaMP6m*.

Both *c164-GCaMP1.3* and *nSyb-GCaMP6m* were then used to cross with mutants of Na^+^ and K^+^ channels genes, e.g., mutants of the *Sh* K^+^ channel (Jan et al., 1977; Wu and Haugland, 1985; Haugland and Wu, 1990), i.e., ortholog of K_V_1 (Iverson et al., 1988; Pongs et al., 1988; Schwarz et al., 1988; Timpe et al., 1988a,b), the *eag* K^+^ channel (Kaplan and Trout, 1969; Ganetzky and Wu, 1983), i.e., EAG (K_V_10, Warmke et al., 1991), and the *para* Na^+^ channel (Suzuki et al., 1971), i.e., Na_V_1 (Loughney et al., 1989).

For the *c164-GCaMP1.3* line, chromosome was then crossed with an attached-X chromosome stock to create the stably maintained strain *X^^^X/+/Y; c164-GCaMP1.3* to generate lines carrying the various mutant alleles of the X-linked Na^+^ and K^+^ channel genes. Male larvae in this attached-X stock, *+/Y; c164-GCaMP1.3* served as the wild-type (WT) control. The mutant genotypes include: i. *Sh^M^*/*Y; c164- GCaMP1.3*, ii. *eag^1^*/*Y; c164-GCaMP1.3*, iii. *eag^1^ Sh^120^*/*Y; c164-GCaMP1.3*, iv. *para^bss1^*/*Y; c164-GCaMP1.3*, v. *para^ts1^*/*Y; c164-GCaMP1.3.* Additional alleles of these genes have been examined to confirm consistent mutational effects, including *Sh^120^* and double mutant *para^bss1^ Sh^120^* (see Results; Table 1).

**Table 1.** Maximum ΔF/F for type I and II synapses of different genotypes

Genotypes	Type Ib		Type Is		Type II	
	40 Hz	2 Hz	10 Hz	20 Hz	2 Hz	10 Hz
	max ΔF/F ± SD (*n*, *N*)	max ΔF/F ± SD (*n*, *N*)	max ΔF/F ± SD (*n*, *N*)	max ΔF/F ± SD (*n*, *N*)	max ΔF/F ± SD (*n*, *N*)	max ΔF/F ± SD (*n*, *N*)
0.1 mM Ca^2+^						
WT	0.16 ± 0.11 (53, 9)	0.07 ± 0.04 (94, 14)	0.08 ± 0.07 (96, 14)	0.43 ± 0.29 (96, 14)	0.12 ± 0.07 (64, 13)	0.57 ± 0.39 (67, 13)
WT + 4AP	1.00 ± 0.33 (18, 3)^***^	0.21 ± 0.12 (32, 4)^**^	0.83 ± 0.33 (32, 4)^***^	1.14 ± 0.41 (32, 4)^***^	0.24 ± 0.15 (21, 4)^**^	1.00 ± 0.40 (21, 4)^***^
WT + TEA	0.91 ± 0.43 (22, 4)^***^	0.12 ± 0.07 (32, 5)^*^	0.12 ± 0.08 (32, 5)^*^	0.86 ± 0.42 (31, 5)^***^	0.71 ± 0.48 (18, 5)^***^	1.02 ± 0.57 (19, 5)^***^
						
*Sh*^*M*^	0.63 ± 0.54 (46, 7)^***^	0.11 ± 0.05 (82, 9)^*^	0.67 ± 0.27 (82, 9)^***^	1.10 ± 0.40 (81, 9)^***^	0.18 ± 0.07 (44, 6)^**^	0.67 ± 0.31 (44, 6)
*Sh*^*120*^	0.18 ± 0.11 (20, 4)	0.08 ± 0.05 (42, 4)	0.63 ± 0.24 (42, 4)^***^	0.99 ± 0.22 (42, 4)^***^	0.18 ± 0.09 (29, 3)^*^	0.64 ± 0.23 (29, 3)
*eag*^*1*^	0.23 ± 0.18 (39, 6)^*^	0.09 ± 0.08 (30, 7)	0.10 ± 0.09 (30, 7)	0.59 ± 0.32 (28, 7)^*^	0.13 ± 0.13 (18, 5)	*0.42 ± 0.37 (20, 5)*
*eag*^*1*^ *Sh*^*120*^	0.59 ± 0.41 (28, 6)^***^	0.43 ± 0.34 (89, 12)^***^	0.83 ± 0.66 (89, 13)^***^	0.89 ± 0.76 (94, 13)^***^	0.17 ± 0.10 (26, 10)^*^	*0.34 ± 0.33 (26, 9)*
						
*para*^*bss1*^	0.22 ± 0.21 (49, 6)	0.07 ± 0.04 (71, 8)	0.35 ± 0.55 (71, 8)^**^	0.64 ± 0.48 (54, 9)^**^	0.46 ± 0.26 (56, 10)^***^	*0.61 ± 0.39 (55, 10)*
*para*^*ts1*^	0.07 ± 0.08 (20, 6)^*^	0.08 ± 0.08 (47, 7)	0.08 ± 0.05 (47, 7)	0.32 ± 0.20 (47, 7)^*^	0.11 ± 0.06 (28, 7)	0.25 ± 0.20 (28, 7)^***^
*para*^*bss1*^ *Sh*^*120*^	0.36 ± 0.23 (35, 6)^***^	0.17 ± 0.13 (35, 6)^**^	0.68 ± 0.46 (35, 6)^***^	0.89 ± 0.64 (35, 6)^***^	0.30 ± 0.24 (22, 5)^***^	*0.61 ± 0.40 (22, 5)*
						
0.5 mM Ca^2+^						
WT	1.11 ± 0.26 (20, 5)^***^	0.12 ± 0.12 (35, 5)^*^	0.43 ± 0.16 (44, 5)^***^	1.22 ± 0.50 (38, 5)^***^	0.33 ± 0.17 (29, 4)^***^	0.85 ± 0.40 (33, 5)^**^
*Sh*^*120*^	**0.93 ± 0.26 (25, 4)**^**+**^	0.11 ± 0.04 (13, 2)	**0.85 ± 0.24 (22, 3)**^**+++**^	1.15 ± 0.19 (22, 3)	0.44 ± 0.23 (31, 4)	**1.23 ± 0.52 (31, 4)**^**+++**^
*para*^*bss1*^	**0.68 ± 0.14 (26, 3)**^**+++**^	0.08 ± 0.04 (47, 4)	0.42 ± 0.28 (49, 4)	1.21 ± 0.55 (48, 4)	**0.73 ± 0.36 (17, 4)**^**+++**^	0.98 ± 0.36 (16, 4)

Data are presented as max ΔF/F ± SD (*n*, *N*), where *n* indicates total bouton number and *N*, NMJ number. N.D., not determined. Bolded numbers are significantly different from WT control (0.1 mM Ca^2+^). Italicized numbers indicate that the ΔF/F traces in the samples (type II) are mostly intermittent. Student’s *t* tests were performed against WT control of the same frequency in 0.1 mM Ca^2+^ concentration (*p < 0.05, **p < 0.01, ***p < 0.001) or in 0.5 mM Ca^2+^ (+*p* < 0.05, ++*p* < 0.01, +++*p* < 0.001).

For *nSyb-GCaMP6m*, virgins of the above *Sh*, *eag* alleles were crossed with male *nSyb-GCaMP6m* to generate Sh^M^/Y; +; *nSyb-GCaMP6m*/+ and *eag^1^*/Y; +; *nSyb-GCaMP6m*/+. RNAi knockdown of *Shab* (*y1 v1; P{TRiP.JF02146}attP2*, Bloomington stock center, see Butler et al., 1989; Tsunoda and Salkoff, 1995; Singh and Singh, 1999; Ueda and Wu, 2006; Peng and Wu, 2007, about *Shab*) was also tested with *nSyb-GCaMP6m*, as specified in figure legends.

The above baseline study were then complemented with additional GCaMP versions, including UAS-*GCaMP6f* (Chen et al., 2013) and UAS*-myrGCaMP5* (Melom and Littleton, 2013; Melom et al., 2013), as well as an additional Gal4 driver *w;;* P{GawB}*386Y* (Wong et al., 2012; Walker et al., 2013). See further details in Results.

### Solutions and preparation

Wandering 3rd instar male larvae were collected from culture bottles and dissected in HL3 saline, containing: 70 mM NaCl, 5 mM KCl, 20 mM MgCl_2_, 10 mM NaHCO_3_, 5 mM trehalose, and 155 mM sucrose; buffered at pH 7.2 with 5 mM HEPES (Stewart et al., 1994). For optical imaging and electrophysiological recording, the saline was replaced with HL3.1 (70 mM NaCl, 5 mM KCl, 4 mM MgCl_2_, 10 mM NaHCO_3_, 5 mM trehalose, 115 mM sucrose, and 5 mM HEPES; at pH 7.2) for reliable detection of the distinct excitability defects previously reported for the Na^+^ and K^+^ mutants (Feng et al., 2004; Ueda and Wu, 2006; Lee et al., 2008). For high pH experiments, Tris (pK_a_ 8.1) was used to replace HEPES in the HL3.1 saline, and the final pH was adjusted with NaOH to 8.8 or 9.8 as specified (Lnenicka et al., 2006). Imaging experiments were performed with saline containing 0.1 mM or 0.5 mM Ca^2+^, as specified. Approximaltey a 5-min equilibration was allowed before stimulation. Ca^2+^ concentration as low as 0.1 mM effectively suppressed muscle contraction, except the occasional occurrence seen in hyperexcitable mutants, e.g., *eag Sh*. Data contaminated with contractions were excluded from further analysis. In experiments with 0.5 mM Ca^2+^, sodium glutamate (7 mM) was added for glutamate receptor desensitization and thus suppression of muscle contraction, a common practice to avoid movement artifacts (Macleod et al., 2002; Reiff et al., 2005; Lnenicka et al., 2006). All of the above chemicals were obtained from Sigma-Aldrich.

### Ca^2+^ imaging

An upright fluorescent microscope (Eclipse E600FN; Nikon) equipped with a 40× water-immersion objective lens (Fluoro; N.A. 0.80) and Nomarski optics was used to visualize synaptic boutons. The light source was a xenon short arc lamp (UXL-75XE; Ushio), filtered by a GFP filter set (excitation filter: 450/50 nm; dichroic mirror: 480 nm; barrier filter: 510/50 nm). Image capture and recording was conducted with the RedshirtImaging NEUROCCD-SM256 system, which includes a CCD camera by SciMeasure Analytical Systems, and the data acquisition control system (Redshirt Imaging). Data compilation and first-order analyses were performed by using the computer software NeuroPlex of the NEUROCCD-SM256 system, which collects both fluorescent images and electrophysiological signals. The digital images (256 × 256 pixels per frame) were sampled at a frame rate of 25 Hz.

The larval segmental nerve bundles were severed from the ventral ganglion. The nerve innervating the hemi-segment monitored for fluorescence was stimulated using a glass suction electrode (∼10 μm in diameter, filled with HL3.1). A second suction electrode (7–8 μm, filled with HL3.1), together with an AC amplifier (GRASS model p15, Warwick, RI), was employed to record resulting action potentials and to determine the stimulation threshold (Wu et al., 1978; Ganetzky and Wu, 1982a), which was usually between 1.5 and 2 V with a stimulus duration of 0.1 ms. The stimulation voltage was set to 4–6 V to ensure action potential initiation.

A GRASS S88 stimulator driven by a programmable pulse generator Master-8 (A.M.P.I.) was used to apply the stimulation protocols. In experiments with 0.1 mM Ca^2+^, 2-, 10-, 20-, 40-, and sometimes 80-Hz stimulation trains (duration 2 s, stimulus pulse width 0.1 ms) were applied sequentially with an inter-trial interval of 4 s (∼2 min for 40- or 80-Hz stimulation). In the experiments with 0.5 mM Ca^2+^, the preparations were first stimulated at 1-Hz for 10 s, followed by 2-s trains of 2-, 5-, 10-, 20-, and 40-Hz stimulation.

### Simultaneous Ca^2+^ imaging and electrical recording

Simultaneous electrophysiological recordings of either nerve action potentials or extracellular focal excitatory junction potentials (efEJP) were sometimes performed during GCaMP Ca^2+^ imaging. A glass electrode (7- to 10-µm opening, filled with HL3.1, 0.1 mM Ca^2+^ and an inserted AgCl-Ag wire) was used for en passant recording of nerve action potentials, as described above, and for efEJP recording, in which the shank of the electrode was heated and bent (∼45–60°). This enabled the electrode tip to approach the muscle surface underneath the objective lens with a steeper angle to form a loose patch covering one to two synaptic boutons (Renger et al., 2000; Ueda and Wu, 2009a, 2012). As demonstrated in Fatt and Katz (1952), extracellular recordings of miniature end-plate potentials (mEPPs) follow the faster time course of miniature end-plate currents (mEPCs) as compared to mEPPs and the registered amplitude is proportional to the local mEPCs as determined by the seal resistance between the electrode tip and muscle membrane (leakage to ground). Signals were recorded with the low and high cutoff frequencies set at 0.1 Hz and 50 kHz and fed to the BNC ports of the NEUROCCD-SM256 system. The digitized signals were processed and stored in a PC computer together with the optical data collected at the same time.

### Pharmacology

Preparations were examined with the standard stimulation protocol in 0.1 mM-Ca^2+^ HL3.1 to obtain control data and subsequently to determine the effects of 4-aminopyridine (4-AP; Sigma-Aldrich), quinidine (Sigma) or tetraethylammonium (TEA; Eastman Kodak). Measured volumes (1–10 µl) of 4-AP, quinidine or TEA stock solutions were added to the bath (∼1 ml) to achieve a final concentration of 200 μM for 4-AP, 20 μM for quinidine, 10 or 20 mM for TEA, or mixtures of these drugs as specified. Gentle pipetting ensured even mixture of the bath solution.

The effects of inhibiting mitochondrial proton gradient was studied with the proton ionophore DNP (Kodak). Dissected larval preparations were first imaged in HL3.1 (0.1 mM Ca^2+^) to obtain control data and the saline was then replaced with HL3.1 containing DNP (0.1 or 0.2 mM, as specified). The effect of DNP incubation was monitored up to 60 min. Sodium azide (NaN_3_; Fisher Scientific), which inhibits the complex VI of electron-transport chain (Bowler et al., 2006), was also tested (1 mM in HL3.1, 0.1 mM Ca^2+^; pH 7.2) using the same protocol.

### Mitochondrial staining

GCaMP-expressing larvae were incubated in HL3.1 saline (0.1 mM Ca^2+^) containing 100 nM tetramethylrhodamine (TMRM; AnaSpec) for 5 min before washing off. An epifluorescence microscope (Eclipse E600FN; Nikon) equipped with a 60× water-immersion lens (Fluoro; N.A. 1.00) was used to collect images from both green (GCaMP) and red (TMRM) channels. GCaMP and TMRM images collected from the same fields were manually merged for optimal superposition of boutons and mitochondria. A custom-made python code was used to threshold the green and red channels separately so as to remove the background and selectively outline boutons and strongly-stained mitochondria.

### Data analysis and statistics

In each larval preparation, data were collected from one or two axonal terminal branches that innervate muscles 12 and 13. For each branch, 3–10 boutons were sampled. Fluorescence intensity at any time point (F_t_) for a bouton was calculated by subtracting the background fluorescence intensity from the bouton fluorescence. The background fluorescence was determined from a selected homogeneous region adjacent to the selected bouton. After background subtraction, baseline fluorescence (F_B_) was calculated from the mean of the read-out in the 25 frames (1-s duration) before the beginning of stimulation. The values of ΔF/F were calculated from (Ft – F_B_)/F_B_. The maximum ΔF/F (max ΔF/F) within the 2-s window following the onset of stimulation was determined following a 5-point running average of the traces collected. The root mean square of the baseline was calculated to indicate the basal noise level (N_B_). Traces with low baseline expression (F_B_) or unusually large noise levels (N_B_) were excluded from analysis (<5% in total). A bouton was considered nonresponding unless the max ΔF/F exceeded 200% of the N_B._ Nonresponding boutons were excluded from kinetics analysis.

The half-rise time (t_1/2Rise_) in kinetic analysis was determined as the time from the onset of stimulation to 50% of the peak value (max ΔF/F) and the half-decay time (t_1/2Decay_) the time period between the end of stimulation and the time point where the signal declined to 50%. (Some mutant larvae showed intermittent Ca^2+^ accumulation, and thus max ΔF/F did not always correspond to the end of stimulation.) All calculations, plots, and pseudo-color maps were constructed with computer programs written with Numpy and Matplotlib packages in Python language (available on request).

**Figure 1.**
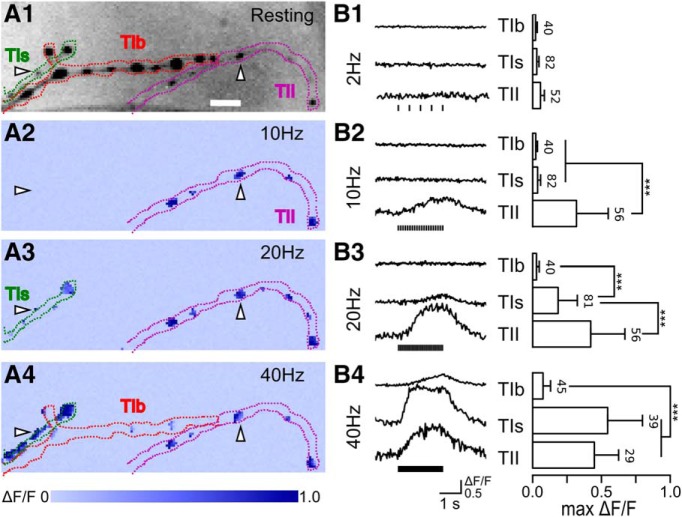
Distinct frequency responses of the GCaMP signals in type Ib, Is, and II synapses. ***A1***, Inverted-gray image showing the typical morphology of GCaMP expressing type Ib, Is, and II synaptic terminals in muscle 12. Their contours are traced in in red, green, and purple, respectively. Arrowheads indicate a type II bouton (right) and a type Is bouton (left). ***A2–A4***, Pseudo-color maps of maximum change in ΔF/F (max ΔF/F) under 10-, 20-, and 40-Hz stimulation, respectively. Arrowheads and terminal contours are carried over from ***A1***. ***B1–B4***, left panels, Representative fluorescence ΔF/F traces and the corresponding right panels show summary bar plots of max ΔF/F for type Ib, Is, and II synaptic boutons from 8–11 NMJs at 2, 10, 20, and 40 Hz, respectively. The vertical scale bar indicates ΔF/F of 0.5 unit, i.e., 50% increase in fluorescence intensity. Error bars indicate SDs with numbers of boutons indicated. KW tests with Bonferroni corrections were performed. Asterisks indicate highly significant differences; ***p < 0.001. Ca^2+^ indicator GCaMP1.3 was used in this and the majority of the following figures, except for Figures 3, 5, 6, 8. These samples are included in the analysis of a greater data set presented in Table 1.

Means and SEMs of data grouped by NMJs are shown in all figures, except for Figure 1, where means and SDs of all boutons for each type of synaptic terminals are shown to indicate the extent of variability. In addition, means and SDs for all datasets are reported in Tables 1–3. For datasets that are normally distributed, either *t* test or one-way ANOVA and Fisher’s LSD *post hoc* tests are used to determine significant differences between means. Data sets that failed to pass the normality tests, were subjected to Kruskal–Wallis (KW) tests with Bonferroni corrections for statistical differences (as specified in figure legends and Tables 1–3, as well as in the statistics table, Table 4). Statistic tests were performed using OriginPro 9.0 made by OriginLab (http://www.originlab.com), Microsoft Excel or custom-made python code.

**Table 4.** Statistics table

	**Data structure**	**Type of test**	**Power**
Figure 1B2	Not assumed^*^	KW tests with Bonferroni correction^**^	TII vs TIb, *p* = 6.60 × 10^−15^ TII vs TIs, *p* = 6.31 × 10^−18^
Figure 1B3	Not assumed	KW tests with Bonferroni correction	TII vs TIs, *p* = 6.31 × 10^−8^ TIs vs TIb, *p* = 3.40 × 10^−13^
Figure 1B4	Not assumed	KW tests with Bonferroni correction	TII vs TIb, *p* = 5.90 × 10^−13^ TIs vs TIb, *p* = 1.41 × 10^−14^
Figure 2C	Not assumed	KW tests with Bonferroni correction	10 Hz, TII vs TIb, *p* = 0.000835 10 Hz, TII vs TIs, *p* = 3.08 × 10^−5^ 20 Hz, TII vs TIb, *p* = 0.000835 20 Hz, TIs vs TIb, *p* = 0.00706 40 Hz, TII vs TIb, *p* = 0.000835 40 Hz, TIs vs TIb, *p* = 0.00706
Figure 2D	Not assumed	KW tests with Bonferroni correction	2 Hz, TII vs TIb, *p* = 0.0628 10 Hz, TIs vs TIb, *p* = 0.0489 10 Hz, TII vs TIb, *p* = 0.0271 20 Hz, TIs vs TIb, *p* = 0.0489 40 Hz, TIs vs TII, *p* = 0.0628
Figure 4B	Not assumed	KW tests with Bonferroni correction	TIs, *eag Sh* vs control, *p* = 2.78 × 10^−5^ TIs, 4-AP vs control, *p* = 0.0177 TII, TEA vs control, *p* = 0.00942 TII, *eag Sh* vs control, *p* = 0.0302
Figure 4D	Not assumed	KW tests with Bonferroni correction	TII, TEA vs control, *p* = 0.131 TIs, 4-AP vs control, *p* = 0.0177 TIs, *Sh* vs control, *p* = 0.00228 TIs, *eag Sh* vs control, *p* = 4.29 × 10^−5^
Figure 10B	Not assumed	KW tests with Bonferroni correction	TII, 2 Hz, *bss1* vs control, *p* = 0.0337 TII, 10 Hz, *para* vs control, *p* = 0.0441 TII, 20 Hz, *para* vs control, *p* = 0.0335
Figure 13B	Normal	*t* tests	TIb, 80 Hz, pH 8.8 vs pH 7.2, *p* = 0.00665 TIs, 40 Hz, pH 8.8 vs pH 7.2, *p* = 0.00222 TIs, 80 Hz, pH 8.8 vs pH 7.2, *p* = 0.000524 TII, 10 Hz, pH 8.8 vs pH 7.2, *p* = 0.000585 TII, 20 Hz, pH 8.8 vs pH 7.2, *p* = 0.0283 TII, 40 Hz, pH 8.8 vs pH 7.2, *p* = 0.0232
Figure 13C	Normal	*t* tests	TIb, 80 Hz, pH 8.8 vs pH 7.2, *p* = 0.00665 TIs, 40 Hz, pH 8.8 vs pH 7.2, *p* = 0.0463
Figure 13D	Normal	*t* tests	TIb, 40 Hz, pH 8.8 vs pH 7.2, *p* = 0.00665 TII, 10 Hz, pH 8.8 vs pH 7.2, *p* = 0.0358
Figure 13E	Normal	ANOVA and Fisher’s LSD tests	Across all boutons and frequencies, Group a vs group b and c, *p* < 0.001 Group b vs group c, *p* < 0.05
Figure 14D	Not assumed	KW tests with Bonferroni correction	TIb, 20 Hz, 60 vs 20 min, *p* = 0.013 TIb, 40 Hz, 60 vs 20 min, *p* = 8.78 × 10^−9^ TIb, 40 Hz, 60 min vs NoDNP, *p* = 9.11 × 10^−9^ TIs, 10 Hz, 60 vs 20 min, *p* = 7.54 × 10^−7^ TIs, 20 Hz, 60 min vs NoDNP, *p* = 3.12 × 10^−8^ TIs, 20 Hz, 60 vs 20 min, *p* = 0.00206 TIs, 40 Hz, 60 min vs NoDNP, *p* = 2.43 × 10^−8^ TIs, 40 Hz, 60 vs 20 min, *p* = 6.14 × 10^−8^ TII, 10 Hz, 20 min vs NoDNP, *p* = 0.0126 TII, 20 Hz, 20 min vs NoDNP, *p* = 1.34 × 10^−7^ TII, 40 Hz, 20 min vs NoDNP, *p* = 2.61 × 10^−10^
Figure 14E	Not assumed	KW tests with Bonferroni correction	TIb, 40 Hz, 60 min vs NoDNP, *p* = 7.24 × 10^−5^ TIb, 40 Hz, 60 vs 20 min, *p* = 4.39 × 10^−4^ TIs, 20 Hz, 60 min vs NoDNP, *p* = 0.00141 TIs, 40 Hz, 60 min vs NoDNP, *p* = 0.000198 TII, 20 Hz, 20 min vs NoDNP, *p* = 7.23 × 10^−7^ TII, 40 Hz, 20 min vs NoDNP, *p* = 7.86 × 10^−8^
Figure 14F	Not assumed	KW tests with Bonferroni correction	TIb, 10 Hz, 60 min vs NoDNP, *p* = 2.66 × 10^−4^ TIb, 10 Hz, 60 vs 20 min, *p* = 8.05 × 10^−5^ TIb, 20 Hz, 60 vs 20 min, *p* = 2.02 × 10^−8^ TIb, 20 Hz, 60 min vs NoDNP, *p* = 1.65 × 10^−8^ TIb, 20 Hz, 20 min vs NoDNP, *p* = 2.23 × 10^−9^ TIb, 40 Hz, 60 vs 20 min, *p* = 8.88 × 10^−4^ TIb, 40 Hz, 60 min vs NoDNP, *p* = 9.80 × 10^−10^ TIb, 40 Hz, 20 min vs NoDNP, *p* = 1.27 × 10^−9^ TIs, 10 Hz, 60 min vs NoDNP, *p* = 4.15 × 10^−5^ TIs, 10 Hz, 20 min vs NoDNP, *p* = 8.53 × 10^−6^ TIs, 20 Hz, 60 min vs NoDNP, *p* = 0.000114 TIs, 20 Hz, 20 min vs NoDNP, *p* = 0.00312 TII, 40 Hz, 20 min vs NoDNP, *p* = 1.83 × 10^−11^
Figure 15	Normal	*t* tests with Bonferroni correction	TIb vs TIs, *p* = 0.044 TIb vs TII, *p* = 3.73 × 10^−13^ TIs vs TII, *p* = 9.46 × 10^−6^

* KW tests do not assume that the data structure is normal.

** All *p* values involving Bonferroni correction have been multiplied with the number of comparisons within the same group of data, whereas the significance levels are set at 0.05, 0.01, and 0.001 for clarity of display in the figures.

## Results

### Distinct frequency responses of GCaMP Ca^2+^ signals in glutamatergic type Ib, Is, and aminergic type II synapses

We performed a comprehensive Ca^2+^ imaging analysis by targeted expression of GCaMP indicators in the larval NMJ and found striking differences in the ionotropic glutamatergic type Ib (tonic) and type Is (phasic), and metabotropic octopaminergic type II synapses. Most Ca^2+^ imaging studies have used abdominal muscles 6 and 7 (Macleod et al., 2002; Reiff et al., 2005; Lnenicka et al., 2006), which are innervated by types Ib and Is, but not by type II, motor axon terminals. We instead chose muscles 12 and 13 as the primary regions of interest. These two muscles are innervated by all three types of synapses, which are individually identifiable by distinct synaptic bouton sizes and terminal branches (Fig. 1*A1*), as shown previously in immunostaining studies (Johansen et al., 1989; Budnik et al., 1990; Kurdyak et al., 1994; Monastirioti et al., 1995; Zhong and Wu, 2004; Koon et al., 2011). Type Ib, Is, and II axonal terminals each represent the projection from a different motor neuron. Notably, the phasic type Is synaptic terminals in muscles 6, 7, 12, and 13 are derived from separate axonal branches of a single motor neuron (MNSNb/D-Is; Hoang and Chiba, 2001; see also Lnenicka and Keshishian, 2000), whereas muscles 12 and 13 share the same type II motor neuron input (MNSNb/D-II; Hoang and Chiba, 2001; see also Schmid et al., 1999). However, the tonic type Ib motor terminals in muscle 12 and 13 are separately innervated by different motor neurons (MN12-Ib and MN13-Ib; Hoang and Chiba, 2001).

The motor patterns found in *Drosophila* larval NMJs usually consist of trains of high-frequency repetitive action potentials (Budnik et al., 1990; Cattaert and Birman, 2001; Fox et al., 2006; Chouhan et al., 2010). To explore how axonal firing frequency determines the GCaMP Ca^2+^ signals in different synaptic boutons, we applied 2-s trains of repetitive stimuli to the motor axons at increasing frequencies (2, 10, 20, and 40 Hz), which are within the normal range of firing rates of type Ib and Is motor axons (Cattaert and Birman, 2001; Chouhan et al., 2010).

We used HL3.1 saline (Feng et al., 2004; Ueda and Wu, 2006) to optimize the expression of the well characterized excitability mutant phenotypes while retaining a desirable property of the HL3 saline (Stewart et al., 1994) of promoting the longevity of the NMJ preparation. Except for some experiments examining Ca^2+^ dependence, the majority of experiments were conducted with saline containing 0.1 mM Ca^2+^, which not only could enhance the hyperexcitable mutant phenotypes but also effectively suppressed muscle contraction during imaging (cf. Ueda and Wu, 2009a,b; Ueda and Wu, 2015). This approach also circumvented the use of high-concentration glutamate for postsynaptic receptor desensitization to suppress muscle contraction during imaging (Macleod et al., 2002, 2004, 2006; Lnenicka et al., 2006) and thus minimized potential complications from activating presynaptic metabotropic glutamate receptors (Zhang et al., 1999).

At low frequencies of nerve stimulation (e.g., 2 Hz), we did not detect any significant GCaMP Ca^2+^ signals (ΔF/F) in WT larvae (Fig. 1*B1*). With 10-Hz stimulation, only type II synapses displayed GCaMP signals (see the pseudo-color map in Fig. 1*A2*, and example traces with bar graphs in Fig. 1*B2* ). GCaMP signals appeared in type I synapses only at higher stimulation frequencies, above 20 Hz for type Is synapses (Fig. 1*A3*,*B3* ) and beyond 40 Hz for type Ib synapses (Fig. 1*A4*,*B4* ).

The distinct frequency responses of GCaMP Ca^2+^ signals in type Ib, Is, and II boutons described above apparently reflect intrinsic differences in synaptic properties and are also evident for NMJs in other ventral and dorsal muscles examined. As demonstrated in Figure 1*A*, despite the large variations in size and fluorescence intensity (Fig. 1*A1*), the boutons along the same terminal branch behaved relatively uniformly (Fig. 1*A2–A4* ) and consistent in the general wave form (see representative traces in Fig. 1*B1–B4*), so that clear distinction in the threshold of frequency responses could be established among each type II, Is, and Ib motor terminals. More detailed morphometric analyses of Ca^2+^ fluorescent signals demonstrate that the characteristic frequency responses of type Ib, Is, and II synapses were independent of and could not be attributed to differences in basal GCaMP expression levels or synaptic bouton sizes (Xing and Wu, 2016).

**Figure 2.**
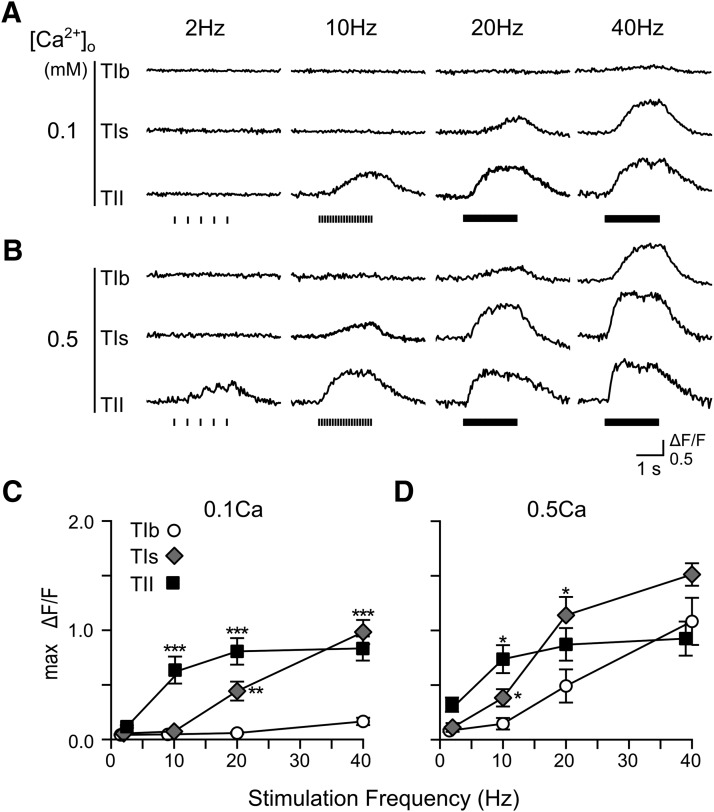
Frequency responses of GCaMP1.3 Ca^2+^ signals at different extracellular Ca^2+^ concentrations. ***A***, ***B***, Example ΔF/F traces for type Ib, Is, and II synaptic boutons stimulated at 2, 10, 20, and 40 Hz in 0.1 and 0.5 mM Ca^2+^ saline. ***C***, ***D***, Summary line plots showing the frequency responses of averaged max ΔF/F for type Ib, Is, and II synapses at 0.1 mM Ca^2+^ (***C***; 8–11 NMJs, same set of data as in Fig. 1 replotted for comparison) and 0.5 mM Ca^2+^ (***D***; four NMJs). In this and Figures 4, 10, 13, for each NMJ, one synaptic terminal was measured for each type of motor synapses and readings of all boutons within the same terminal were averaged. See Table 1 for total bouton numbers and corresponding NMJ numbers. For clarity, some data points are displaced horizontally and statistically significant differences against type Ib synapses are indicated at each stimulus frequency. In this figure and Figures 4, 10, 13, 14, error bars indicate SEM. Asterisks denote levels of statistically significant differences (*p < 0.05; **p < 0.01; ***p < 0.001), based on KW tests and Bonferroni correction.

We found that the distinction in frequency responses among type Ib, Is, and II synapses remained at higher Ca^2+^ concentrations (with 7 mM glutamate in HL3.1 to suppress muscle contraction; Macleod et al., 2004), with the same sequence of responsiveness to various stimulation frequencies. As shown in Figure 2, at 0.5 mM external Ca^2+^, type Ib synapses remained to be the least responsive and type II the most responsive (20 Hz for type Ib and 2 Hz for type II; Fig. 2*B*,*D*), despite the overall enhanced fluorescent signals compared to 0.1 mM Ca^2+^ (Fig. 1*B1–B4* ). Consistently, the data also indicate that saturation levels of GCaMP signals were reached at different frequencies for three types of boutons (at 0.5 mM Ca^2+^, 10 Hz for type II, ∼40 Hz for type Is, and well above 40 Hz for type Ib; Fig. 2*B*,*D*).

**Figure 3.**
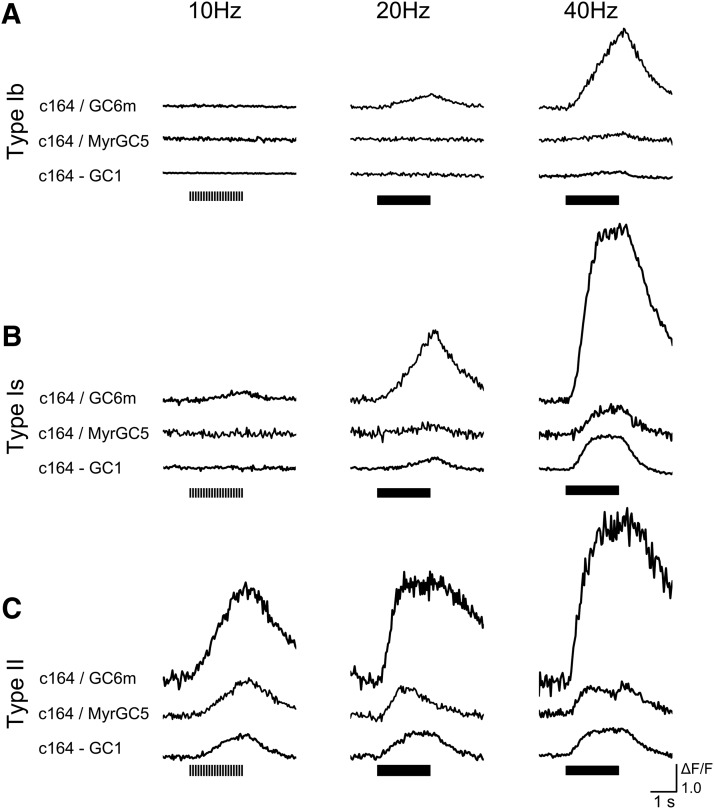
Signals generated by GCaMP indicators of different sensitivity and subcellular localization in response to 10-, 20-, and 40-Hz stimulation. ***A****–****C***, Example ΔF/F traces for type Ib, Is, and II synaptic boutons recorded with different indicators, c164/GC6m, c164/myrGC5, and c164-GC1 (standing for genotypes: *c164-Gal4*/*UAS-GCaMP6m*, *c164-Gal4*/+; *UAS-myrGCaMP5*/+ and +/Y; *c164-GCaMP1.3*), in 0.1 mM Ca^2+^ saline at the designated stimulation frequencies. Consistently, GCaMP signals of type II synapses appeared in the lowest and type Ib the highest stimulus frequency ranges, regardless of the indicator types.

The relative differences in GCaMP signal frequency responses remained unaltered for the various GCaMP indicators with different sensitivity and subcellular localization. Importantly, as Figure 3 shows, similar distinctions in frequency responses were obtained among the three synaptic types with myrGCaMP5 (Melom and Littleton, 2013; dissociation constant *K*_d_ = 447 nM, Akerboom et al., 2012), GCaMP6m (*K*_d_ = 167 nM; Chen et al., 2013), and GCaMP1.3 (*K*_d_ = 234 nM; Nakai et al., 2001), despite their differences in affinity and sensitivity. Additional indicator GCaMP6f and the Gal4 driver *c386Y* produced the same frequency responses. In particular, MyrGCaMP5, which is localized to plasma membrane (Melom and Littleton, 2013), still produced the same frequency response differences, suggesting that surface-volume ratio differences among type Ib, Is, and II synaptic boutons did not significantly contribute to their distinct GCaMP signal frequency dependencies, which was consistent with previous report on mammalian neurons (Mao et al., 2008).

### Differential excitability control of residual Ca^2+^ dynamics by K^+^ channels in type Ib, Is, and II synapses

Previous work has shown that neuronal hyperexcitability caused by mutations or drug blockage of the various K^+^ channels could lead to greatly enhanced transmitter release often associated with different patterns of repetitive firing of axonal action potentials at *Drosophila* larval NMJs (Ganetzky and Wu, 1982a, 1983, 1985; Ueda and Wu, 2006). Since GCaMP Ca^2+^ imaging enabled detection at a subcellular resolution to indicate variations in excitability properties among the three categories of synapses, we examined the effects of altering specific K^+^ channels pharmacologically. The differentiation power was further enhanced by systematic analyses of mutants of identified K^+^ channels (Table 1).

**Figure 4.**
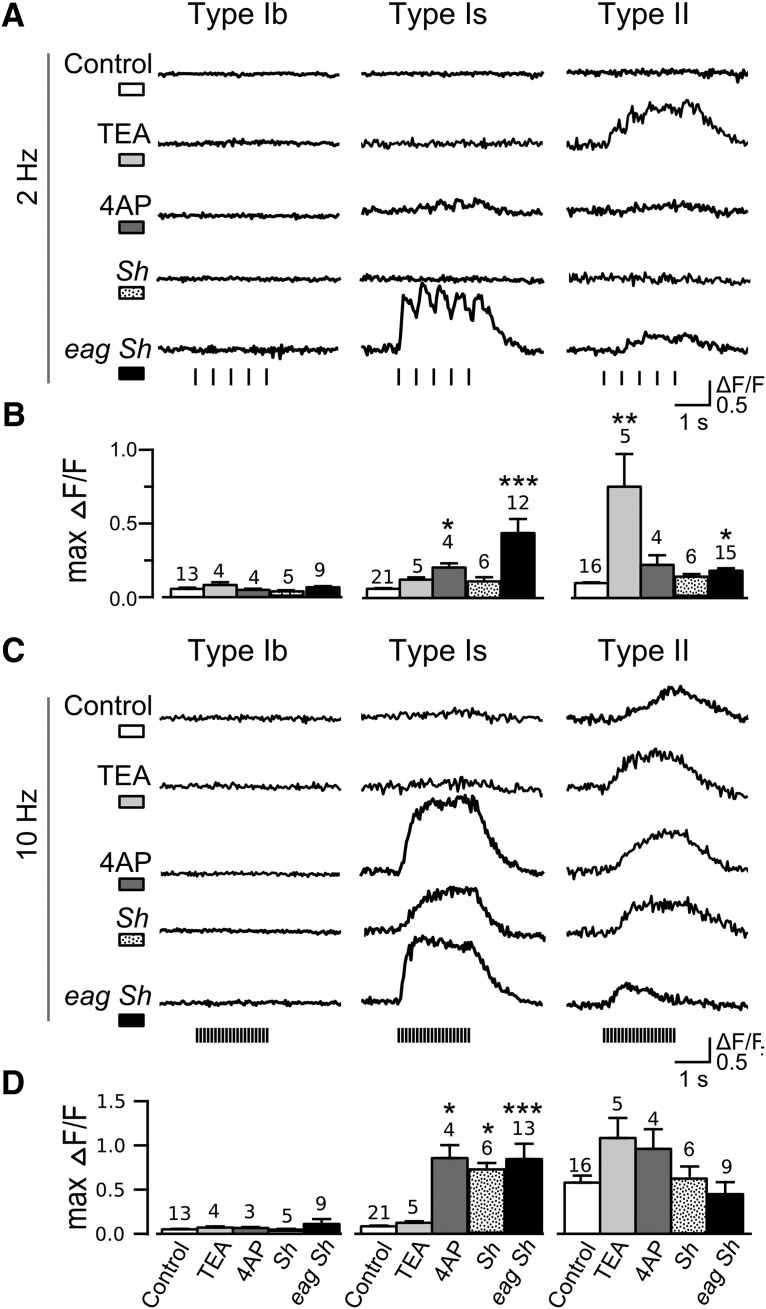
Genetic and pharmacological dissection of the distinct excitability of type Ib, Is, and II synapses. Representative ΔF/F traces under 2- and 10-Hz stimulations (***A***, ***C***), comparing WT control, WT with TEA (10 mM) and 4-AP (200 μM) treatments, *Sh^M^* mutant and double mutant *eag^1^ Sh^120^*. Note the preferential effects of TEA on type II, and 4-AP, *Sh^M^* and *eag^1^ Sh^120^* on type Is synapses. ***B***, ***D***, Bar charts for sample statistics corresponding to the traces in ***A***, ***C***, with NMJ numbers and SEMs indicated. KW tests with Bonferroni correction were performed; *p < 0.05, **p < 0.01, ***p < 0.001.

TEA and 4-AP are two well-characterized, commonly used K^+^ channel blockers known to cause neuronal hyperexcitability in *Drosophila* NMJs (Jan et al., 1977; Ganetzky and Wu, 1983, 1985; Fox et al., 2005; Ueda and Wu, 2006), as well as other species (Thesleff, 1980; Hille, 2001). Both TEA (10 mM) and 4-AP (200 μM) treatments shifted the frequency responses so that substantial GCaMP Ca^2+^ signals appeared at lower stimulus frequencies (Table 1, compare WT with 4-AP and TEA rows). However, as shown in Figure 4, striking preferential effects were apparent, with TEA strongly enhancing GCaMP signals in type II (at frequencies as low as 2 Hz; Fig. 4*A*,*B*) and 4-AP greatly increasing that in type Is synapses (10 Hz; Fig. 4*C*,*D*). For type Ib synapses, the effects of TEA and 4-AP were milder and only evident at high stimulation frequencies (above 40 Hz; Table 1). Notably, *Sh* mutations (*Sh^M^*, null allele, *Sh^120^*, point mutation) and 4-AP closely resemble in their differential effects on type Ib, Is, and II synapses (Fig. 4; Table 1), consistent with the fact that 4-AP specifically blocks the *Sh* K_V_1 channels that mediate fast-inactivating transient K^+^ current I_A_ in muscle (Salkoff and Wyman, 1983; Wu and Haugland, 1985). Unlike 4-AP, TEA has a different and wider spectrum of action on several types of K^+^ channels at tens of millimolar concentrations (Koketsu, 1958; MacKinnon and Yellen, 1990; Hille, 2001). The contrasting preferential effects of 4-AP on type Is and TEA on type II raise the possibility of different K^+^ channel compositions in type Ib, Is, and II synapses.

One of the most striking demonstrations of extreme hyperexcitability in *Drosophila* NMJs can be seen in *eag Sh* double mutants, in which axon bundles display high-frequency (∼100 Hz) repetitive firing of supernumerary action potentials triggered by just one nerve stimulus (Ganetzky and Wu, 1982a, 1983, 1985; Wu et al., 1983). However, it has not been resolved how type Is and Ib terminals each contributes to this extreme phenotype. As shown in Figure 4, in the double-mutant *eag^1^ Sh^120^*, type Is was clearly the most drastically affected, since robust single stimulus-evoked GCaMP signals could be observed only in type Is, but not Ib, synapses (Fig. 4*A*, 2 Hz), and could not be seen in either *Sh* or *eag* single mutants (Table 1). Enhanced GCaMP signals in type Ib were seen only when stimulated beyond 40 Hz (Table 1, first column) while type II synapses only occasionally displayed single stimulus-evoked responses (see further details below). Taken together, these observations demonstrate type Is motor axon activities as the source of the supernumerary repetitive axonal action potentials in *eag Sh* double mutants and support the notion of different combinations of K^+^ channel subtypes in type Ib, Is, and II synapses.

**Figure 5.**
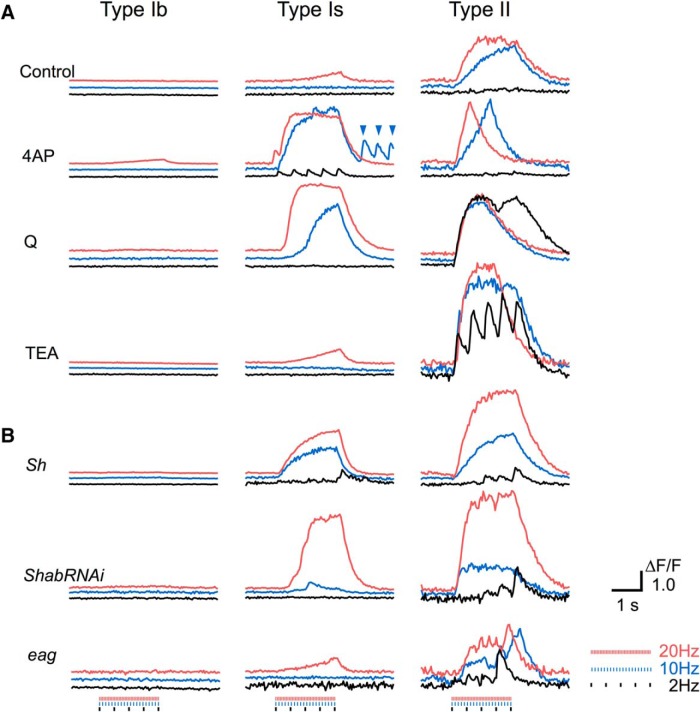
Distinct effects of various genetic and pharmacological perturbations of K^+^ channels on type Ib, Is, and II synapses at different stimulus frequencies. Note that these perturbations preferentially lowered the frequency threshold for GCaMP signals in type Ib, Is, and II synapses. ***A***, +; +; *nSyb-GCaMP6m* larvae used as WT control (0.1 mM Ca^2+^, top-row traces), examined by applying 4-AP (200 µM), quinidine (abbreviated as Q, 20 µM), and TEA (10 mM). Black trace, 2-Hz stimulation; blue, 10 Hz; red, 20 Hz. Note the drastic frequency response change in type II synapses (2 Hz, Q and TEA), in type Is (10 Hz, 4-AP), and in type Ib (20 Hz, 4-AP). ***B***, Genetic perturbation of GCaMP signals examined with *Sh^M^/*Y;; *nSyb-GCaMP6m*/+, *eag^1^/*Y;+; *nSyb-GCaMP6m/*+, and *UAS-ShabRNAi/+*; *nSyb-GCaMP6m*/+. Three to five larvae for each condition were tested and consistent results were obtained.

**Figure 6.**
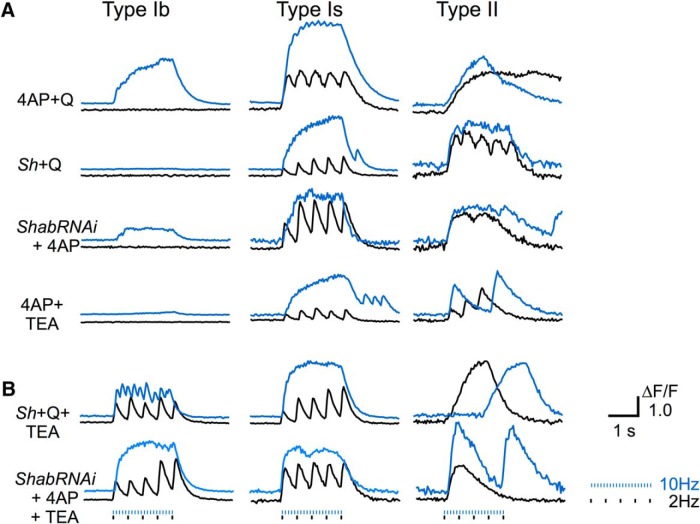
Extreme excitability indicated by single pulse-evoked giant GCaMP signals: double and triple insults of combined mutation and drug effects. GCaMP6m signals (ΔF/F) in response to 2-Hz (black) and10-Hz (blue) stimulation from larvae carrying the *nSyb-GCaMP6m* construct (0.1 mM Ca^2+^). *nSyb-GCaMP6m*, used as WT control (0.1 mM Ca^2+^), examined by coapplication ***A***, Double insults of combined effects of 4-AP (200 µM) + quinidine (Q, 20 µM), *Sh^M^* + quinidine (20 µM), *ShabRNAi* + 4-AP (200 µM), and 4-AP (200 µM) + TEA (10 mM). Note the robust GCaMP signals in type II and Is synapses evoked by individual stimuli (2 Hz) and the preferential response of type Ib synapses to combined insults of 4-AP + Q or *ShabRNAi* (10 Hz). ***B***, Triple insults of K^+^ channel perturbations with *Sh^M^* + Q (20 µM) + TEA (10 mM), or *ShabRNAi* + 4-AP (200 µM) + TEA (10 mM). Note the single pulse-evoked giant GCaMP signals in type Ib synapses (2 Hz), indicating a state near the ceiling effect of hyperexcitability. Also note that hyperexcitability resulted in interrupted, aborted or rebounding GCaMP signals in type II synapses (2 and 10 Hz) and rebounds of type Is responses (10 Hz). Based on observations from three to five larvae for each condition.

To extend the above findings based on GCaMP1.3 measurements, we performed a separate set of experiments with GCaMP6m, an improved version of GCaMP with increased sensitivity (compare Fig. 3), for a more systematic analysis of the distinctions in K^+^ channel functioning among type Ib, Is, and II synapses (Figs. 5, 6).

For the ease of comparisons, representative traces of GCaMP6 responses to 2-, 10-, and 20-Hz stimulation are superimposed for each genotype and condition. For the cases of altered type II synapses, mutations of *eag* channels, as well as *ShabRNAi* knockdown or quinidine (abbreviated as Q in all figures) inhibition of *Shab* K_V_2 channels (Singh and Wu, 1989; Wu et al., 1989; Singh and Singh, 1999; Ueda and Wu, 2006), led to clearly discernable GCaMP responses to individual nerve stimuli delivered at 2 Hz in HL3.1 saline containing 0.1 mM Ca^2+^ (Fig. 5; 2-Hz traces). Even more robust effects were obtained with TEA treatment, which has a broad-spectrum action and can act on *Shab* and *eag* channels (Pak et al., 1991; Brüggemann et al., 1993). However, type II synaptic GCaMP signals were less affected by *Sh* mutations or 4-AP treatment, consistent with the GCaMP1.3 results (Fig. 4; Table 1).

In comparison, type Is synapses were most strongly influenced by manipulations of *Sh* channels, with some small GCaMP responses to individual stimuli in the 2-Hz stimulus train detectable in *Sh* or 4AP-treated WT NMJs (Fig. 5, middle column). For type Ib synapses, however, none of the above genetic or pharmacological manipulations was effective in producing GCaMP responses with stimulus frequencies below 20 Hz (Fig. 5, middle column). These GCaMP6m observations confirm the conclusions drawn from the experiments based on GCaMP1 (Fig. 4; Table 1) regarding the differential excitability control of by K^+^ channels in type Ib, Is, and II synapses. Taken together, it could be seen that type II synapses were most sensitive to manipulations of *eag* and *Shab* channels, whereas type Is synapses appeared to be particularly vulnerable to disruptions of *Sh* channels, and type Ib synapses were most resistant to K^+^ channel perturbations.

### Extreme hyperexcitability and motor terminal repetitive firing

Apparently, a ceiling effect of hyperexcitability is indicated by the robust responses to individual stimuli (delivered in the 2-Hz nerve stimulation), as seen in TEA-treated type II synapses (Fig. 5, right column). We found that type Is synapses could also reach this ceiling effect at 0.1 mM Ca^2+^ but only after double insults of the combined perturbations of *Sh* with either *eag* or *Shab* channels. Similar to the GCaMP1 results of *eag Sh* double mutants (Fig. 4), greatly enhanced GCaMP6m signals in response to individual nerve stimuli could be seen in type Is synapses by the joint effects of 4AP (or *Sh* mutations) with either quinidine (or *ShabRNAi*) or TEA (Fig. 6*A*, middle column, 2-Hz traces).

In contrast, type Ib synapses remained largely nonresponsive at 2 Hz during the above double perturbations (Fig. 6*A*, left column). It required at least 10 Hz for type Ib synapses to exhibit clearly detectable GCaMP signals even with both *Sh* and *Shab* channels disrupted (Fig. 6*A*; for single perturbation results, see Table 1; Fig. 5). However, type Ib synapses could display the hallmark of hyperexcitability if given the condition of triple insults with mutations or blockers of K^+^ channels. With triple insults, i.e., combinations of TEA and 4AP (or *Sh*) plus quinidine (or *ShabRNAi*), type Ib synapses exhibited single pulse-evoked robust GCaMP signals in 0.1 mM Ca^2+^ saline (Fig. 6*B*, left column, 2-Hz traces), directly comparable to the ceiling level of hyperexcitability found in type Is and type II synapses (Figs. 5, 6*A* ). Therefore, compared to type II and Is synapses, type Ib synapses appeared to possess a greater K^+^ channel-mediated repolarization capacity and were hence more resilient to disruptions in different types of K^+^ channels.

**Figure 7.**
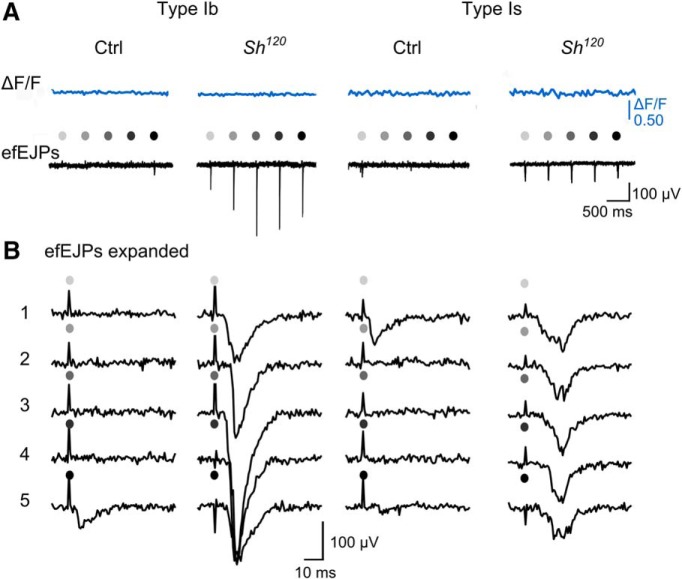
Failure to detect presynaptic GCaMP signals when synaptic transmission events were registered by efEJPs. ***A***, Traces of GCaMP1.3 ΔF/F (blue) and efEJPs (black) at 2-Hz stimulation collected from the same boutons of type Ib and Is motor terminals of WT control (*c164-GCaMP1.3*) and *Sh^120^/*Y; *c164-GCaMP1.3*. Each stimulus is indicated by a gray-scaled dot. ***B***, Expanded traces of efEJPs in response to the sequential stimuli that are shown in ***A***. Note that no detectable GCaMP signals (***A***) despite significant transmissions (***B***).

**Figure 8.**
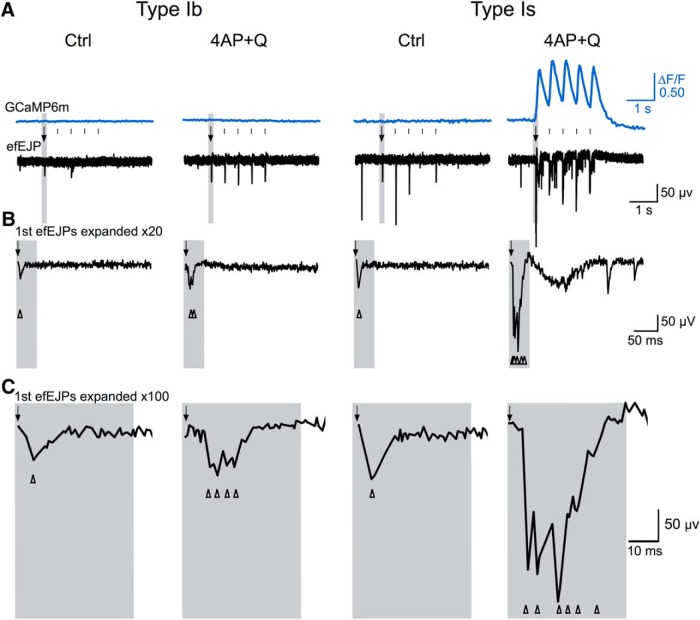
Correlation of giant GCaMP signals with repetitive efEJPs evoked by individual stimuli. ***A***, ΔF/F signals of the GCaMP6m indicator correlated with simultaneous focal recording of efEJPs from the same boutons in type Ib and Is motor terminals (2-Hz stimulation in 0.1 mM Ca^2+^ HL3.1 saline). K^+^ channel blockade with 4-AP (200 µM) and quinidine (Q, 20 µM) in *nSyb-GCaMP6m* larvae led to giant GCaMP signals in type Is, but not Ib, synapses. Expanded efEJP traces (20 and 100 times, shaded time windows) in ***B***, ***C*** indicate supernumerary transmitter releases (arrowheads) following each nerve stimulus (arrow).

To understand how the modified GCaMP signals were correlated with the amplitude and frequency of the synaptic transmission events, we performed simultaneous focal recording in conjunction with GCaMP imaging on the same synaptic boutons. efEJPs follow the faster time course of excitatory junctional currents (EJCs) as compared to EJPs and their amplitudes are linearly proportional to the local EJCs generated by the synaptic release in region under the electrode tip (Fatt and Katz, 1952; Sakmann and Neher, 1984). This approach enabled a direct correlation of postsynaptic response induced by transmitter release with the presynaptic GCaMP signals triggered by nerve stimulation. At low external Ca^2+^ (0.1 mM), both type Ib and Is produced only small efEJP responses at a level of quantal fluctuation in WT larvae and there were no detectable signals from GCaMP1.3 and GCaMP6m indicators (Figs. 7, 8). However, we found both GCaMP1.3 and GCaMP6m indicators were unable to produce detectable signals even when efEJPs were clearly discernable. For example, type Ib and Is boutons in *Sh* larvae (Fig. 7), or in WT following 4-AP and quinidine treatment (Fig. 8), produced greatly enhanced efEJPs that were not registered by either GCaMP1.3 or GCaMP6m.

**Figure 9.**
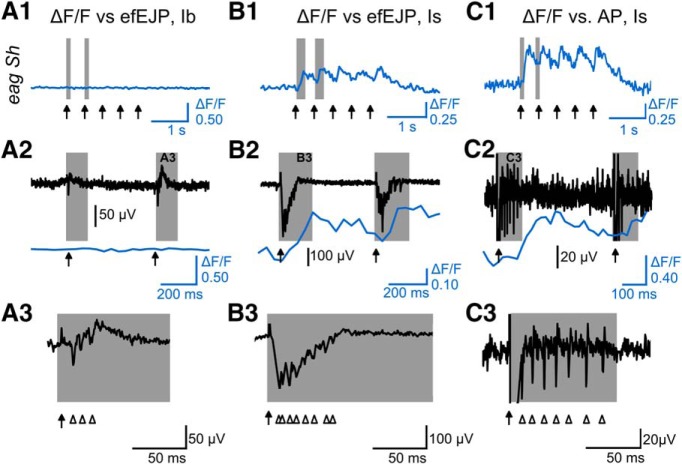
Electrophysiological basis of single pulse-evoked giant GCaMP signals: repetitive firing due to extreme hyperexcitability of the motor axon. GCaMP signals (ΔF/F traces) of type Ib (***A1***) and Is (***B1***, ***C1***) synapses from three *eag^1^ Sh^120^*/Y; *c164-GCaMP1.3* larvae at 2-Hz stimulation in 0.1 mM Ca^2+^ HL3.1 saline. Corresponding simultaneous records of efEJPs (***A2***, ***B2***) and nerve action potentials (***C2***) are displayed in black with the associated ΔF/F traces in blue (expanded from ***A1***, ***B1***, ***C1*** with individual nerve stimuli marked by arrows). One of the two shaded areas in ***A2***, ***B2***, ***C2*** are further expanded to show efEJPs with multiple peaks (arrowheads), indicative of repetitive nerve action potentials, which were more robust in type Is than Ib synapses (***A3***, ***B3***). En passant nerve recording revealed motor axon high-frequency repetitive firing (over 100 Hz; ***C3***).

A notable exception was during the robust 2-Hz peaks of GCaMP6 signals (the hallmark hyperexcitability ceiling effect) in type Is synapses at low external Ca^2+^ (0.1 mM) after double insults of K^+^ channels, such as application of 4-AP plus quinidine, where giant efEJPs occurred in correlation with each GCaMP ΔF/F peak (Fig. 8, right column; compare Fig. 6, middle column,). A closer examination of these highly nonlinear events revealed that these efEJPs consisted of massive discharge of multiple release of transmitters (expanded type Is efEJP traces in Fig. 8; and also Fig. 9 for *eag Sh* double mutant effect). Further investigation with triple recordings to correlate axonal action potentials with efEJP and GCaMP signals demonstrated that these massive efEJP discharges underlying individual GCaMP ΔF/F peak were evoked by high-frequency supernumerary firing of motor axons (∼100 Hz; Fig. 9).

It is important to note that in all of the experiments reported here, the segmental nerve was severed from the ventral ganglia. Therefore, the repetitive action potentials were generated within the axon and were not driven by CNS activities mediated by the motor neurons. Thus, these hallmark, ceiling effects of GCaMP signals reflect axonal membrane hyperexcitability and are consistent with previous reports of multiple firing of motor axons associated with prolonged, giant EJPs at low external Ca^2+^ levels found in *eag Sh* (Ganetzky and Wu, 1982a) and *Sh Shab* (Ueda and Wu, 2006) double mutants.

### Differential effects of Na^+^ channel mutations on GCaMP signals and nerve conduction failure in type II motor axons

The above observations reveal distinct repolarization mechanisms of the three synaptic terminals, presumably reflecting differential expression of K^+^ channel genes. We found similarly disparate sensitivities to Na^+^ channel mutations among the three types of synapses, although in *Drosophila para* is the only known gene that encodes voltage-gated Na^+^ channel (Na_V_1), which regenerates a large number of splice variants (Loughney et al., 1989; Thackeray and Ganetzky, 1994; Olson et al., 2008; Lin et al., 2012). To investigate the role of the Na^+^ channel, we took advantage of two well-characterized *para* alleles that confer drastic but opposite effects on the membrane depolarization mechanism. The hypoexcitable, temperature-sensitive allele *para^ts1^* is thought to decrease the synthesis of functional Na^+^ channels (Thackeray and Ganetzky, 1994), resulting in increased axonal refractory period and higher sensitivity to Na^+^ channel-specific toxins (Wu and Ganetzky, 1980; Suzuki and Wu, 1984), and becomes paralyzed at high temperature due to axonal conduction failure (Suzuki et al., 1971; Siddiqi and Benzer, 1976; Ganetzky et al., 1986). In contrast, the hyperexcitable, bang-sensitive allele *bss1* displays seizure behaviors on mechanical stress (Ganetzky and Wu, 1982b; Burg and Wu, 2012), which is associated with seizure-like nerve spike discharges in central circuits (Lee and Wu, 2006; Parker et al., 2011) and increased synaptic transmission with axonal multiple firing at the larval NMJ (Ganetzky and Wu, 1982b; Giachello and Baines, 2015).

**Figure 10.**
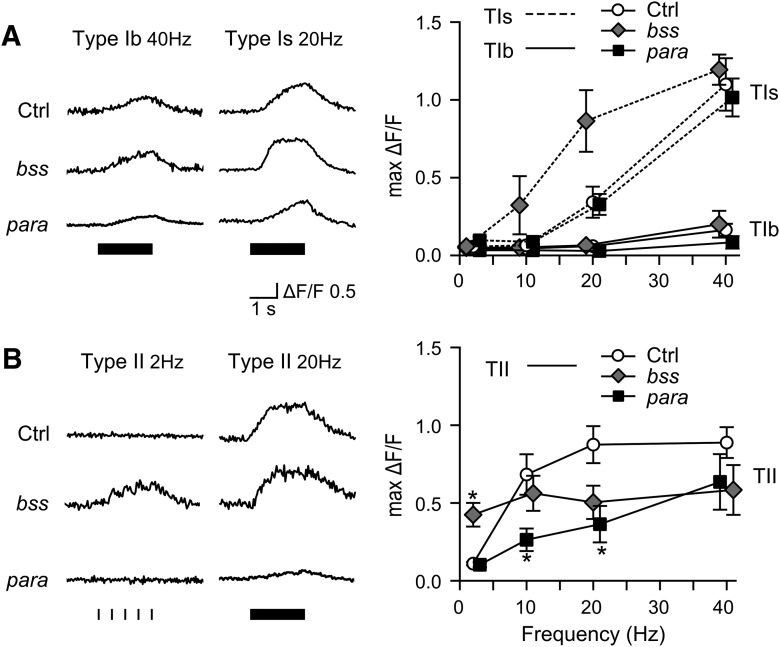
Differential effects of Na^+^ channel mutations on the Ca^2+^ dynamics of type Ib, Is, and II synapses. GCaMP Ca^2+^ signals of type Ib and Is (***A***) and II (***B***) NMJs from a hypoexcitable allele, *para^ts1^* (*para*) and a hyperexcitable allele, *para^bss1^* (*bss*) are compared, with representative ΔF/F traces at specified frequencies shown in left panels and the corresponding frequency dependence of max ΔF/F in right panels (mean and SEM, of 6–11 NMJs for each genotype, compare Table 1). Note that type II synaptic boutons in *bss* frequently displayed “intermittent” or “aborted” GCaMP signals on repetitive stimulation, lowering the average max ΔF/F above 10 Hz (see Fig. 11 for details). KW ANOVA with Bonferroni corrections were performed (*p < 0.05, **p < 0.01).

The GCaMP1 imaging demonstrated that like *Sh* K^+^ channel mutations, the hyperexcitable *para* allele *bss1* had a strong preferential effect on type Is over Ib synapses (Fig. 10*A*, 10 and 20 Hz; Table 1). Notably, GCaMP signals were significantly enhanced in type Is boutons on stimulation at 10 and 20 Hz, whereas little effect on type Ib synapses was detectable up to 40 Hz. This preferential effect again raises the possibility of differential expression or posttranslational modifications of Na^+^ channel slice isoforms (Thackeray and Ganetzky, 1994; Olson et al., 2008; Lin et al., 2012). However, the *bss* effect on type Ib could be revealed once combined with *Sh^120^* since the double mutant produced much larger response than either single mutant alone (Table 1). On the contrary, in both type Ib and Is synapses, the hypoexcitable temperature-sensitive *para* allele *ts1* produced only small, but statistically significant, reductions in the GCaMP signals at room temperature (Table 1; Fig. 10*A*).

**Figure 11.**
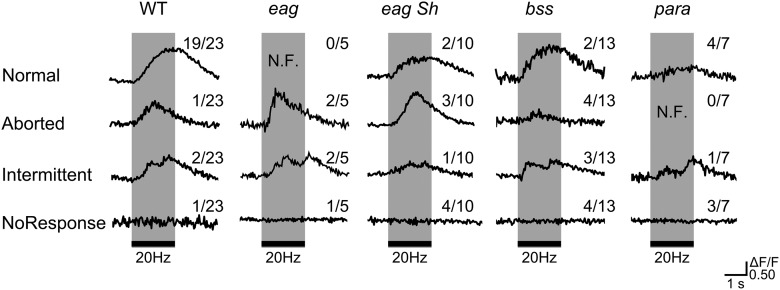
Variability in the wave form of GCaMP signals in type II synaptic terminals at high frequencies of stimulation. Representative regular (normal) and irregular (aborted, intermittent, and no response) ΔF/F traces at 20 Hz from WT, *eag*, *eag Sh*, *bss*, and *para* type II boutons (with *c164-GCaMP1.3*) are shown. For each genotype, the numbers of NMJs exhibiting each wave form are indicated together with the total sample size (numbers of NMJs examined). N.F., not found. Note that both hypoexcitable (*para^ts1^*) and hyperexcitable (others) mutations increased the occurrence of irregular waveforms, indicating axonal conduction failure in type II motor terminals (see text). Similar response waveforms were also revealed by GCaMP6m indicator (e.g., for *eag^1^*/Y;; *nSyb-GCaMP6m*/+; normal: 0/6, aborted: 2/6, intermittent: 3/6, no response: 1/6).

In comparison, type II synapses in both *ts1* and *bss1* alleles produced more striking, clear-cut alterations. The hypoexcitable *ts1* mutation results in significantly smaller GCaMP signals in type II synapses even at room temperature (10 and 20 Hz; Fig. 10*B*). Notably, a significant fraction of the stimulation trials produced no response at all (20-Hz sample trace in Fig. 10*B*), indicating axonal conduction failure. This lowered safety margin for action potential propagation in the type II motor axons could be explained by the higher longitudinal internal resistance for axons with smaller diameters (Aidley, 1998), making them more vulnerable to the effect of reduced density or modified function in Na^+^ channel that determine the depolarizing Na^+^ current across the plasma membrane. Such axonal conduction failure was not observed in the larger type Ib or Is motor terminals in *para^ts1^* NMJs. For the hyperexcitable allele *bss1*, a striking nonlinear frequency response was illustrated by the GCaMP signals in type II synapses. Greatly enhanced GCaMP signals were seen at 2 Hz, with discernable responses to individual stimuli (compare type II, 2-Hz traces in Figs. 5, 10*B*). However, at increased stimulus frequencies a suppression of GCaMP responses was seen, concurrently with a novel, unexpected pattern of interrupted or aborted GCaMP signals. These events occurred in the rising phase of GCaMP signals during repetitive stimulation (Fig. 11), resulting in a net decrease in the average Max ΔF/F in *bss1* (Fig. 10*B*, plot, 20–40 Hz). Boutons of the same type II synaptic terminal in the same NMJ behaved uniformly in the interrupted or aborted pattern, with the timing and kinetics entirely the same. Such seemingly puzzling GCaMP responses suggest intermittent axonal AP conduction failure at increasing stimulus frequencies.

It has been shown that in the oocyte expression system, *para^bss1^* Na^+^ channels exhibit slower recovery from inactivation (Parker et al., 2011), which could lengthen action potential refractory period. This would make small-diameter type II axons particularly prone to action potential failure at high stimulation frequencies.

It is important to point out that a similarly phenomenon of interrupted GCaMP signals in type II motor terminals was also found in other hyperexcitable K^+^ channel mutants, such as *eag* and *eag Sh* (Fig. 11; see also Fig. 4, *eag^1^ Sh^120^* type II, 10 Hz). This apparently counter-intuitive observation in hyperexcitable K^+^ channel mutants could also be associated with increased action potential refractory period (cf. *para^bss1^*), which made the axons nonresponsive to the upcoming stimuli after excessive firing activities. It is known that in *eag^1^ Sh^120^* a longer refractory period (Engel and Wu, 1992) results from excessive inactivation of Na^+^ channels following over excitation due to weakened repolarizing K^+^ currents. As shown in Figures 5, 6, different mutational and drug manipulations of K^+^ channels could lead to irregular waveforms of GCaMP6 signals, most pronouncedly in type II synapses. Intermittent, delayed, aborted, or sometimes lost accumulation of the GCaMP signal occurred frequently (Fig. 5, type II 10 and 20 Hz). Following more drastic manipulations (double or triple insults), these irregularities became more extreme, occurring even at 2-Hz stimulation (Fig. 6). Notably, these extreme hyperexcitability conditions also caused GCaMP signal rebound after stimulus cessation, which was encountered more frequently in type II and occasionally in type Is synaptic terminals (Figs. 5, 6).

### Differential effects on rise and decay kinetics of alterations in Ca^2+^ influx and clearance

The above irregular waveforms of GCaMP signals reflect either high-frequency axonal repetitive firing or intermittent axonal conduction failure. However, in most cases GCaMP signals registered gradual residual Ca^2+^accumulation and followed a time course of smooth rise during repetitive stimulation and an approximately exponential decay at the cessation of stimulus trains.

**Figure 12.**
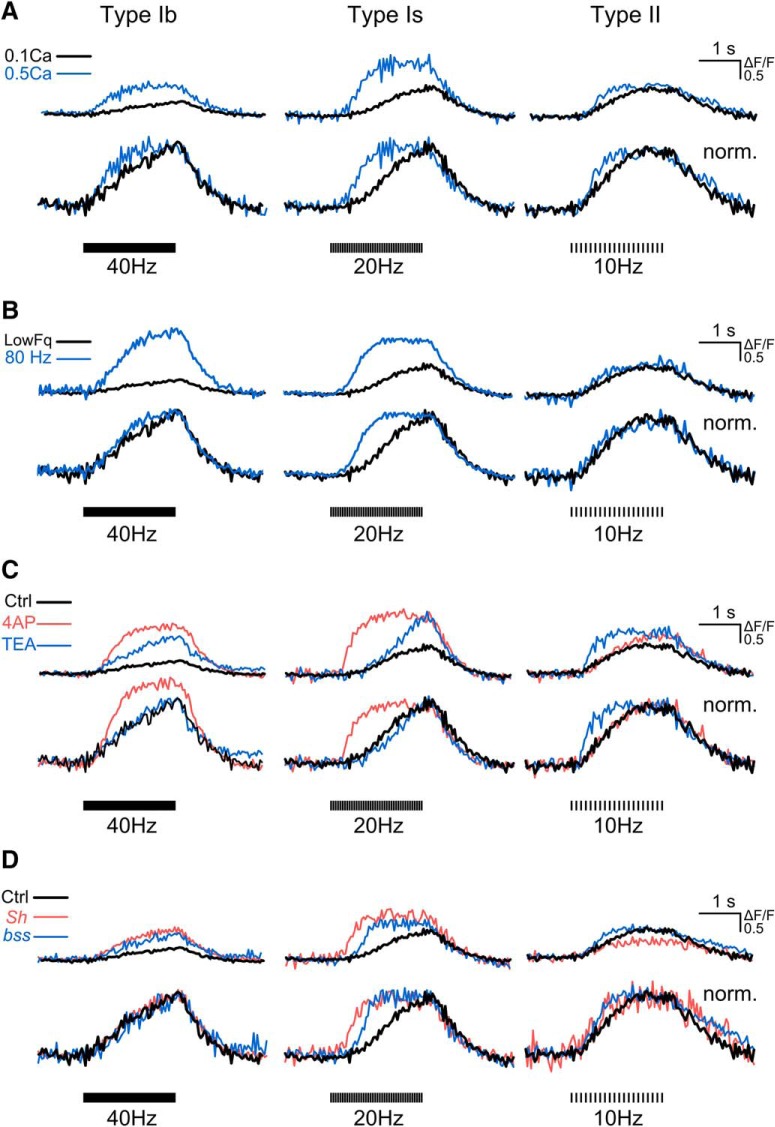
Kinetic analysis of presynaptic GCaMP signals with different manipulations enhancing Ca^2+^ influx. Comparison of original (upper) and normalized (lower) ΔF/F traces collected from individual boutons of (***A***) WT with 0.1 mM Ca^2+^ (black) versus 0.5 mM Ca^2+^ (blue), (***B***) WT with 0.1 mM Ca^2+^ under the designated stimulation frequency (black, 40 Hz for Ib, 20 Hz for Is, and 10 Hz for II) versus 80-Hz stimulation (blue), and (***C***) WT control (black) versus 4-AP (200 µM, red) and TEA (10 mM, blue) treatments, 0.1 mM Ca^2+^. ***D***, WT control (black) versus *Sh^M^* (*Sh*, red), and *bss1* (*bss*, blue). Type Ib, Is, and II synapses, stimulated at 40, 20, and 10 Hz, respectively, in 0.1 mM Ca^2+^. Note clear alterations in the rise kinetics with minimal effects in the decay kinetics indicated by superimposing normalized traces. See Tables 2, 3 for ensemble data of rise and decay kinetics.

In principle, the GCaMP Ca^2+^ signal reflects an integrated dynamic process of Ca^2+^ influx and subsequent clearance of intracellular free Ca^2+^. Thus, analysis of the rise and decay kinetics of GCaMP signals may yield further insight into how alterations in membrane excitability can change the time course of intracellular Ca^2+^ accumulation. To visualize the overall GCaMP Ca^2+^ signal rise and decay kinetics in response to repetitive stimulation, ΔF/F were normalized and overlaid to contrast for changes caused by channel blockers and mutations (Fig. 12; Tables 2, 3). As expected, we found that the rise phase of GCaMP signals was accelerated by acute application of higher external Ca^2+^ concentrations (Fig. 12*A*), increased stimulus frequencies (Fig. 12*B*), K^+^ channel blockers (Fig. 12*C*), and mutations that increase membrane excitability (Fig. 12*D*). All of these manipulations, including 4-AP and TEA treatments as well as *Sh* and *bss1* mutations, resulted in faster GCaMP signals with shortened peaking or plateauing time. All these changes in rise kinetics were indicated by shortened half-rise time, as quantified and summarized in Table 2. Note that the preferential effects of individual channel blockers and mutations on type Ib, Is, and II synapses are again illustrated in the extents of rise time acceleration (Fig. 12). Single pulse-evoked GCaMP signals showed even shorter peaking time (Figs. 5, 6), consistent with the high-frequency repetitive firing of action potentials triggered by each stimulus pulse in these cases of hyperexcitability (Fig. 9).

**Table 2.** Rise kinetics for type I and II synapses of different genotypes

	Type Ib	Type Is		Type II		
Genotype	40 Hz	20 Hz	40 Hz	10 Hz	20 Hz	40 Hz
	t_1/2Rise_ ± SD (*n*, *N*)	t_1/2Rise_ ± SD (*n*, *N*)	t_1/2Rise_ ± SD (*n*, *N*)	t_1/2Rise_ ± SD (*n*, *N*)	t_1/2Rise_ ± SD (*n*, *N*)	t_1/2Rise_ ± SD (*n*, *N*)
0.1 mM Ca^2+^						
WT	1.12 ± 0.26 (18,5)	1.17 ± 0.19 (99, 14)	0.68 ± 0.21 (47, 9)	0.94 ± 0.32 (68, 13)	0.67 ± 0.20 (72, 13)	0.68 ± 0.20 (24, 7)
WT + 4AP	0.66 ± 0.10 (19, 3)^***^	0.39 ± 0.09 (32, 4)^***^	0.34 ± 0.17 (29, 3)^***^	0.76 ± 0.18 (20, 4)^**^	0.48 ± 0.24 (20, 4)^**^	0.49 ± 0.32 (21, 3)^**^
WT + TEA	1.03 ± 0.23 (20, 4)	1.00 ± 0.22 (29, 4)^**^	0.61 ± 0.13 (25, 4)	0.43 ± 0.27 (15, 5)^***^	0.55 ± 0.30 (15, 5)^*^	0.55 ± 0.19 (19, 4)
						
*Sh*^*M*^	0.89 ± 0.21 (47, 7)^**^	0.43 ± 0.12 (81, 9)^***^	0.39 ± 0.27 (67, 9)^***^	0.85 ± 0.25 (41, 6)^*^	0.62 ± 0.16 (43, 6)	0.51 ± 0.22 (29, 6)^**^
*Sh*^*120*^	1.20 ± 0.13 (13, 3)	0.49 ± 0.21 (35, 4)^***^	0.23 ± 0.14 (23, 4)^***^	0.89 ± 0.19 (23, 3)^*^	0.58 ± 0.14 (26, 3)^*^	N.D.
*eag*^*1*^	1.10 ± 0.22 (31, 6)	1.12 ± 0.29 (28, 6)	0.64 ± 0.14 (56, 6)	N.A.^†^	N.A.^†^	N.A.^†^
*eag*^*1*^ *Sh*^*120*^	0.78 ± 0.23 (25, 6)^***^	0.39 ± 0.23 (55, 7)^***^	0.25 ± 0.13 (46, 5)^***^	N.A.^†^	N.A.^†^	N.A.^†^
						
*para*^*bss1*^	1.10 ± 0.18 (39, 9)	0.74 ± 0.38 (65, 9)^***^	0.63 ± 0.14 (29, 3)	N.A.^†^	N.A.^†^	N.A.^†^
*para*^*ts1*^	1.02 ± 0.26 (6, 3)	1.28 ± 0.19 (26, 5)^*^	0.75 ± 0.20 (33, 7)	N.A.^††^	N.A.^††^	0.87 ± 0.31 (20, 3)
*para*^*bss1*^ *Sh*^*120*^	1.19 ± 0.17 (27, 4)	0.33 ± 0.06 (33, 6)^***^	N.D.	0.61 ± 0.34 (11, 5)^**^	**0.53 ± 0.16 (22, 6)** ^***^	N.D.
						
0.5 mM Ca^2+^						
WT	0.53 ± 0.19 (25, 4)^***^	0.57 ± 0.12 (31, 5)^***^	0.28 ± 0.06 (34, 4)^***^	0.43 ± 0.17 (33, 5)^***^	0.36 ± 0.21 (36, 5)^***^	0.23 ± 0.13 (29, 5)^***^
*Sh*^*120*^	0.62 ± 0.07 (25, 3)	**0.33 ± 0.11 (22, 3)**^**++**^	**0.18 ± 0.05 (17, 2)**^**+++**^	**0.52 ± 0.14 (32, 4)**^**++**^	0.27 ± 0.09 (33, 4)	0.23 ± 0.15 (27, 4)
*para*^*bss1*^	**0.69 ± 0.12 (26, 3)**^**++**^	**0.58 ± 0.10 (48, 4)**^**+++**^	0.25 ± 0.06 (34, 3)	**0.28 ± 0.15 (18, 4)**^**+++**^	**0.21 ± 0.09 (19, 4)**^**+++**^	0.26 ± 0.14 (12, 4)

Data are presented as t_1/2Rise_ ± SD (*n*, *N*), where t_1/2Rise_ indicates half-rise time in seconds. N.A., not applicable, because of intermittent responses (†) or low-amplitude signals masked by baseline noise (††). N.D., not determined. The bolded font indicates significant difference from wild-type control. Student’s *t* tests were performed against WT control at the same frequency in 0.1 mM Ca^2+^ (*p < 0.05, **p < 0.01, ***p < 0.001) or in 0.5 mM Ca^2+^ (+*p* < 0.05, ++*p* < 0.01, +++*p* < 0.001).

**Table 3.** Decay kinetics for type I and II synapses of different genotypes

	Type Ib	Type Is		Type II		
Genotype	40 Hz	20 Hz	40 Hz	10 Hz	20 Hz	40 Hz
	t_1/2Decay_ ± SD (*n*, *N*)	t_1/2Decay_ ± SD (*n*, *N*)	t_1/2Decay_ ± SD (*n*, *N*)	t_1/2Decay_ ± SD (*n*, *N*)	t_1/2Decay_ ± SD (*n*, *N*)	t_1/2Decay_ ± SD (*n*, *N*)
0.1 mM Ca^2+^						
WT	0.53 ± 0.19 (18,5)	0.46 ± 0.20 (102, 16)	0.50 ± 0.11 (47, 8)	0.77 ± 0.27 (67, 13)	0.84 ± 0.20 (72, 13)	0.73 ± 0.20 (24, 6)
WT + 4AP	0.52 ± 0.07 (19, 3)	0.48 ± 0.10 (32, 4)	0.53 ± 0.09 (29, 3)	0.85 ± 0.24 (20, 4)	0.74 ± 0.23 (18, 4)	1.07 ± 0.23 (18, 4)^***^
WT + TEA	0.46 ± 0.10 (17, 4)	0.56 ± 0.23 (29, 4)^*^	0.56 ± 0.15 (25, 4)	0.64 ± 0.25 (16, 5)	0.64 ± 0.37 (16, 5)	0.88 ± 0.29 (19, 4)
						
*Sh*^*M*^	0.40 ± 0.08 (47, 7)^*^	0.52 ± 0.10 (81, 9)^*^	0.48 ± 0.11 (67, 8)	0.81 ± 0.25 (37, 6)	0.77 ± 0.39 (29, 6)	0.71 ± 0.25 (21, 6)
*Sh*^*120*^	0.40 ± 0.10 (13, 3)	0.56 ± 0.11 (35, 4)^*^	0.54 ± 0.09 (23, 4)	0.78 ± 0.19 (23, 3)	0.72 ± 0.24 (26, 3)^*^	N.D.
*eag*^*1*^	0.58 ± 0.32 (31, 6)	0.47 ± 0.20 (28, 6)	0.60 ± 0.13 (56, 5)^*^	N.A.^†^	N.A.^†^	N.A.^†^
*eag*^*1*^ *Sh*^*120*^	0.54 ± 0.23 (25, 6)	0.68 ± 0.30 (65, 10)^*^	0.57 ± 0.20 (46, 5)	N.A.^†^	N.A.^†^	N.A.^†^
						
*para*^*bss1*^	0.42 ± 0.19 (39, 7)^*^	0.55 ± 0.09 (58, 7)	0.48 ± 0.09 (29, 6)	N.A.^†^	N.A.^†^	N.A.^†^
*para*^*ts1*^	N.A.^††^	0.49 ± 0.20 (26, 5)	0.48 ± 0.13 (33, 7)	N.A.^††^	N.A.^††^	0.70 ± 0.24 (20, 4)
*para*^*bss1*^ *Sh*^*120*^	0.64 ± 0.45 (27, 4)	0.50 ± 0.20 (33, 6)	N.D.	0.76 ± 0.23 (18, 6)	0.83 ± 0.36 (22, 6)	N.D.
						
0.5 mM Ca^2+^						
WT	0.45 ± 0.08 (20, 4)	0.47 ± 0.10 (35, 5)	0.50 ± 0.05 (38, 4)	0.71 ± 0.36 (33, 5)	0.89 ± 0.37 (37, 5)	0.60 ± 0.20 (28, 4)
*Sh*^*120*^	0.41 ± 0.09 (25, 3)	0.48 ± 0.09 (22, 3)	0.55 ± 0.10 (17, 2)	0.93 ± 0.25 (32, 4)	0.90 ± 0.36 (33, 4)	0.78 ± 0.34 (27, 4)
*para*^*bss1*^	0.42 ± 0.08 (26, 3)	0.53 ± 0.22 (48, 4)	0.67 ± 0.37 (34, 3)	**1.28 ± 0.41 (18, 4)**^**+++**^	**1.31 ± 0.36 (19, 4)**^**+++**^	**1.13 ± 0.57 (12, 4)**^**+++**^

Data are presented as t_1/2Decay_ ± SD (*n*, *N*), in which t_1/2Decay_ indicates half-decay time in seconds. N.A., not applicable, because of intermittent responses (†) or low-amplitude signals masked by baseline noise (††). N.D., not determined. The bolded font indicates significant difference from wild-type control. Student’s *t* tests were performed against WT control at the same frequency in 0.1 mM Ca^2+^ (*p < 0.05, **p < 0.01, ***p < 0.001) or in 0.5 mM Ca^2+^ (+*p* < 0.05, ++*p* < 0.01, +++*p* < 0.001).

Remarkably, when compared to the half-rise time, the half-decay time of GCaMP signals was much less affected by enhanced membrane excitability (4-AP and TEA in Fig. 12*C*; *Sh* and *para^bss1^* in Fig. 12*D*; compare Tables 2, 3 for additional genotypes and conditions). This is true also for a higher external Ca^2+^ concentration (0.5 mM; Fig. 12*A*; Tables 2, 3). These observations apparently reflect an abundant reserve of Ca^2+^ clearance capacities that could still handle the increased influx caused by drug treatments or mutations in our experiments.

However, it should be noted that in extreme hyperexcitability conditions, such as the combined effects of 4-AP, quinidine, and TEA, long-lasting repetitive firing of action potentials could occur after the end of stimulation (Ganetzky and Wu, 1982a; Ueda and Wu, 2006), which extended the decay time course of GCaMP signals in a manner of “rebound” or oscillation (mostly in type II and sometimes in type Is synapses, see examples in Figs. 5, 6).

In addition to manipulating the factors that regulate Ca^2+^ influx, we also examined known mechanisms affecting Ca^2+^ clearance for indications of their differential actions on type Ib, Is, and II synapses. As previously shown for type Ib synapses, a major Ca^2+^ extrusion mechanism in *Drosophila* NMJ is PMCA (Lnenicka et al., 2006; Klose et al., 2009). The H^+^/Ca^2+^ exchange pump PMCA relies on inward transport of proton counter-ions for Ca^2+^export, and can thus be inhibited by high external pH (Niggli and Sigel, 2008). High-pH inhibition of PMCA is effective and has been employed to manipulate Ca^2+^ transients in *Drosophila* NMJ synapses (Lnenicka et al., 2006; Klose et al., 2009; Caldwell et al., 2013), squid giant axon (Dipolo and Beaugé, 1982), and mammalian neurons and pancreatic cells (Benham et al., 1992; Chen et al., 2003).

**Figure 13.**
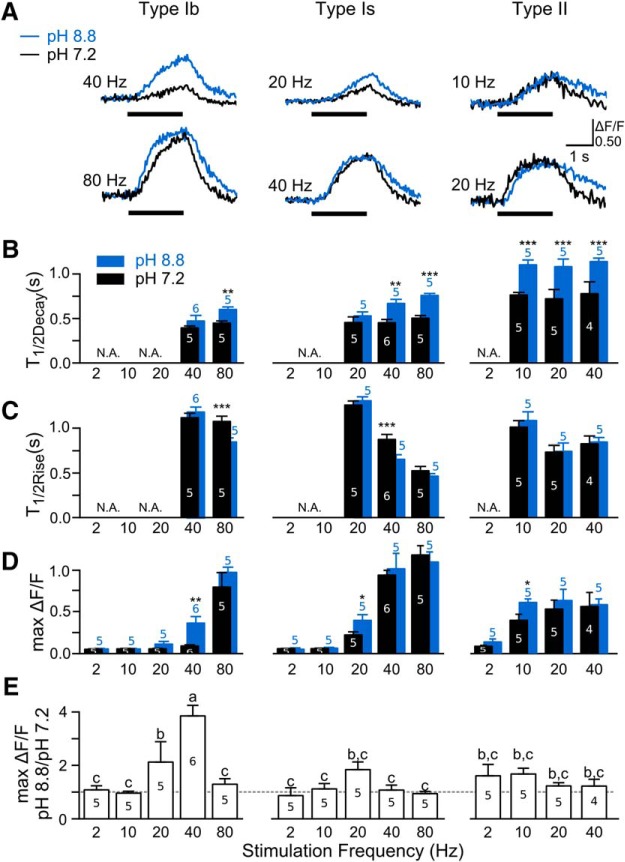
Effects of PMCA inhibition at high pH on Ca^2+^ dynamics of type Ib, Is, and II synapses. ***A***, ΔF/F traces of WT larvae in control pH (7.2, black) and high pH (8.8, blue) saline. Overlapping traces from the same individual boutons illustrate the consequences of suppressing plasma membrane Ca^2+^ extrusion via PMCA at a lower (subplateauing, upper row) and a higher (plateauing, lower row) stimulation frequencies. ***B***–***D***, Averaged half-decay time (T_1/2Decay_), half-rise time (T_1/2Rise_), and peak GCaMP signals (max ΔF/F), respectively, at different stimulation frequencies for type Ib, Is, and II synapses (five to six NMJs from different larvae; N.A., not applicable due to small signals unsuitable for reliable kinetic determination). Statistically significant differences between high and low pH are indicated (*t* tests; *p < 0.05, **p < 0.01, ***p < 0.001). ***E***, Relative increment in GCaMP signal amplitude determined as the ratio of max ΔF/F between pH 8.8 and 7.2 measurements. One-way ANOVA and Fisher’s LSD tests, a, *p* < 0.001, significantly different from group c; b, *p* < 0.05, significantly different from group c. The dashed line indicates the ratio of 1. Note that PMCA inhibition most effectively increased max ΔF/F around the threshold frequencies (***D***, ***E***; i.e., 40, 20, and 10 Hz), coupled with lengthening of decay time above the plateauing frequencies (***B***; i.e., 80, 40, and 10 Hz) for Ib, Is, and II synapses, respectively.

Following an established protocol, we compared Ca^2+^ extrusion efficacy among the three types of synapses in low and high pH saline (pH 7.2 vs 8.8; cf. Lnenicka et al., 2006). The results showed that acutely increased extracellular pH not only slowed down the decay kinetics as expected (Fig. 13*A*,*B*), but could also accelerate the rise and increase the amplitude of GCaMP signals significantly under some conditions (Fig. 13*C–E*).

In pH 7.2 saline, the half-decay time of GCaMP1 signals was ∼0.5 s for both type Ib and Is and ∼0.7–0.8 s for type II synapses at both 0.1 and 0.5 mM Ca^2+^ (Fig. 13*B*; Table 3; other GCaMP indicators reported similar decay times, compare Fig. 3; data not shown). Immediately on pH increase to 8.8, the half-decay time in both type Ib and Is synapses increased to 0.6–0.7 s (Fig. 13*B*, 40–80 Hz) while a more pronounced increase to ∼1.1 s was seen in type II synapses (Fig. 13*B*, 10–40 Hz).

Compared to retarded decay kinetics, high-pH treatment rendered milder effects, in terms of accelerated rise and enhanced peak amplitude, on GCaMP signals in all three types of synapses. Acceleration of the rise phase was detected somewhere below the “saturation frequency” where GCaMP signals approached the highest attainable amplitude (Fig. 13*C*, 80 and 40 Hz for type Ib and Is; compare Figs. 2*C*, 13*D*, Table 1). It is interesting to note that these were similar to the frequencies at which the lengthening in decay time became evident for type Ib and Is synapses (80 and 40 Hz; Fig. 13*B*). In contrast, significant enhancement of amplitude (max ΔF/F) was found around the “threshold frequencies”, where GCaMP signals became readily detectable (Fig. 13*C*, 40, 20, and 10 Hz for type Ib, Is, and II synapses, respectively), and the relative enhancement (fold change) was greatest in type Ib synapses (Fig. 13*D*,*E*, at 40 Hz).

To investigate the extent of involvement of PMCA in synaptic Ca^2+^ clearance, we further increased saline pH to 9.8 and found that pH 9.8 led to greater increases in the decay time of GCaMP signals in all three synapses (>1 s for type I synapses) and shifted GCaMP signals to lower frequency ranges (Ib: 20 Hz, Is: 10 Hz). However, under this condition, the GCaMP signal rapidly ran down within about ten minutes, precluding reliable quantitative determination of the lengthening in half-decay time.

### Prolonged mitochondrial inhibition and energy-dependent Ca^2+^ clearance in type Ib, Is, and II synapses

Exporting Ca^2+^ out of cell or into intracellular organelles against its concentration gradient requires energy expenditure, e.g., ATP hydrolyzis. Mitochondria are critical for ATP synthesis to power active Ca^2+^ clearance mechanisms such as PMCA, a Ca^2+^-ATPase (Zenisek and Matthews, 2000; Shutov et al., 2013). A maintained proton gradient across mitochondrial inner membrane is required for these ATP-dependent Ca^2+^ clearance mechanisms, as well as direct mitochondrial sequestration of intracellular free Ca^2+^ (Tang and Zucker, 1997; David et al., 1998; Suzuki et al., 2002).

**Figure 14.**
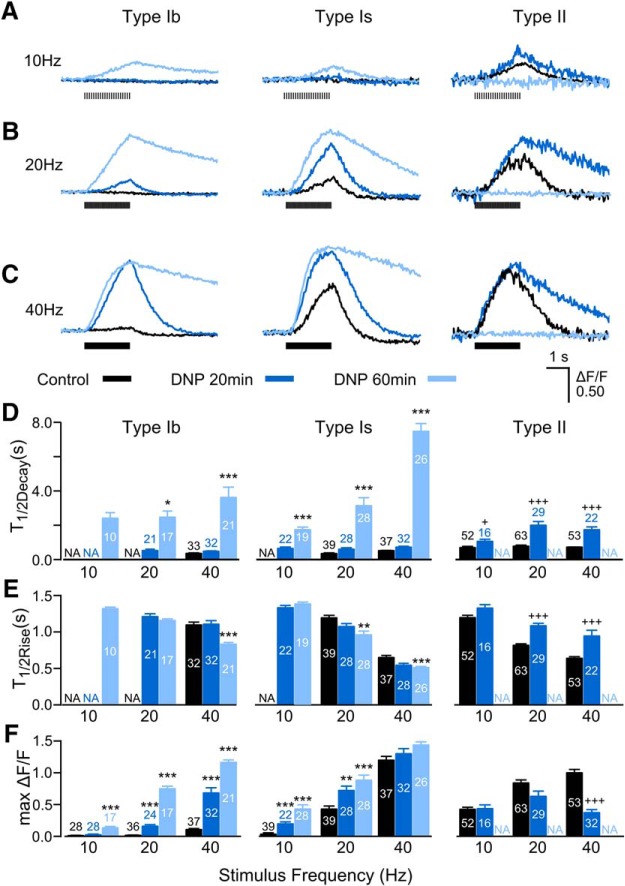
Drastic effects of prolonged DNP treatment on Ca^2+^ dynamics in all three types of synapses. ***A***–***C***, Overlaid representative GCaMP Ca^2+^ signal ΔF/F traces under 10-, 20-, and 40-Hz stimulation, after 0-, 20-, and 60-min exposure to DNP (black, blue, and light blue, respectively). Note that type II synapses (right column) completely lost responses after a 60-min exposure (light blue), and within a 20-min exposure, about half of type II synapses had already lost responses (data not shown). ***D****–****F***, Summary bar charts for the half-decay time, half-rise time, and maximum ΔF/F of GCaMP Ca^2+^ signal under 10, 20, and 40 Hz of repetitive stimulation. N.A., not applicable because the corresponding GCaMP ΔF/F traces were too small or simply nonresponsive to stimulation, thus excluded from kinetic determination (e.g., type II after a 60-min DNP treatment, and type Ib and Is at low stimulation frequencies). Number of boutons (sampled from six different larvae) are indicated. KW tests with Bonferroni corrections were performed among each frequency group; * and +*p* < 0.05, **p < 0.01, *** and +++*p* < 0.001. For type II synapses after a 20-min DNP treatment, + and +++ denote statistical differences with the dwindling sample sizes due to loss of responses in some type II NMJs after DNP treatment.

The proton ionophore DNP is known to reversibly uncouple mitochondrial proton gradient from oxidative phosphorylation (Higgins and Rogers, 1976; Nguyen et al., 1997; Buckler and Vaughan-Jones, 1998; Petrenko et al., 2010). We performed time-lapse measurements of GCaMP signals during the 1-hour DNP treatment. Subsequent to the basal measurements (control, or “0” min; Fig. 14), the preparation was incubated in 0.2 mM DNP saline (0.1 mM Ca^2+^ in HL3.1). Among the three synaptic types, type II synapses first showed drastically lengthened decay time course (20-min incubation; Fig. 14*A–D*, dark blue traces and bars) in correlation with its slowest basal and high pH decay times among the three (Fig. 13*B*; Table 3). On further incubation (up to 60 min), drastically prolonged decay times of GCaMP signals were also observed in type Ib and Is synapses, while type II synapse stopped responding to stimulation by this time (Fig. 14*A–D*, light blue traces and bars). However, further continuous DNP incubation eventually led to total loss of GCaMP responses in all synapses. It is important to note that the lengthened decay time course of individual synaptic terminals was partially reversible, if DNP washout was done before GCaMP response loss. Irreversible loss of GCaMP responses of individual synaptic terminals occurred first in some type II synapses (starting around 20 min into incubation) and then in a smaller portion of type Ib and Is synapses (∼60 min). Compared to the dramatic alterations in decay kinetics, the rise kinetics, i.e., half-rise time, were only mildly affected by DNP treatment (Fig. 14, compare *D*, *E*).

DNP incubation greatly enhanced GCaMP signals of both type Ib and Is synapses so that significantly greater GCaMP signals were observed even at low (10 Hz) stimulation frequencies (Fig. 14*A*,*F*, left and middle columns). Such a shift of threshold frequency was most evident in type Ib synapses (from 40 to 10 Hz; Fig. 14*F*). Notably, DNP effects were further promoted with hyperactivity. It was found that hyperexcitable *eag Sh* accelerated the onset of characteristic DNP effect in type Ib and Is synapses (slower decay and enhanced amplitude) to within 25 min, compared to >40 min in WT. Conceivably, higher activity levels in *eag Sh* could drain the ATP reserve at a faster rate.

A possible effect of long-term proton ionophore (e.g., DNP) treatment is depolarization of the plasma membrane potential (Hodgkin and Keynes, 1955) and thus increased Ca^2+^ influx, as prolonged mitochondrial inhibition leads to diminished ATP supply for Na^+^/K^+^ ATPase, which maintains the resting membrane potential. However, this effect may not have reached a significant level within the time frame of our experiments, i.e., the repetitive firing as demonstrated by correlating focal recording with GCaMP imaging (compare Figs. 8, 9). Within 1 hr, DNP treatments induced enhanced GCaMP signals with prolonged decay time course, but this was seldom accompanied by supernumerary efEJPs or significant elevation of baseline GCaMP fluorescence (except for 1 out of >10 larvae, excluded from analysis). To obtain a different line of evidence for this striking mitochondrial inhibition effect, we used azide, which inhibits the complex IV in the electron transport chain (Fei et al., 2000). Long-term incubation in azide (1 mM, 60–90 min) resulted in similarly drastic slow decay kinetics of GCaMP6m signals (half-decay time >3 s in all three synapses) and shift in threshold frequency (Ib: from 40 to 20 Hz, Is: from 20 to 10 Hz).

**Figure 15.**
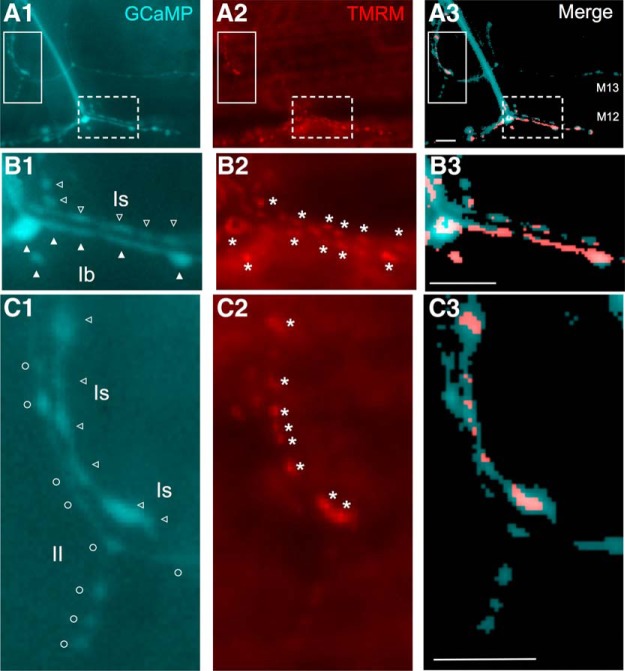
Differential mitochondrial density in type Ib, Is, and II synapses. Columns 1 and 2 are epifluorescent NMJ images obtained with GCaMP1 baseline fluorescence (cyan) and TMRM mitochondrial staining (red), respectively. Column 3 is the merge of columns 1 and 2. The images of the cyan and red channels were separately thresholded before merging to reveal the bouton contour (cyan) and the most strongly stained spots (red). Content in non-bouton, non-axon area was erased in column 3 for clearer demonstration of overlapping. Row ***A*** is representative images of a muscle 12 (bottom, M12 in ***A3***) and 13 (top, M13) NMJs from a WT larva (+/Y; *c164-GCaMP1.3*). The border between M12 and M13 is faintly visible in ***A2***. Rows ***B***, ***C*** are enlarged views of the broken and continuous boxed regions in ***A***, respectively. In Column 1, type Ib, Is, and II boutons are designated with filled arrowheads, open arrowheads, and open circles, respectively. In Column 2, asterisks indicate some areas of stronger TMRM mitochondrial staining. Note that the weak mitochondrial staining in type II synapses was well below the intensity level of type Is synapses. Statistics are presented in Results.

All these lines of evidence indicate that intact mitochondrial metabolism is critical for presynaptic Ca^2+^ clearance, which differs in capacity in type Ib, Is, and II synapses. We therefore examined the mitochondrial density in these synapses by staining the NMJ with TMRM, a fluorescent indicator sequestered into mitochondria by the electrochemical proton gradient (Scaduto and Grotyohann, 1999). By overlaying the mitochondrial staining with GCaMP fluorescence, it was evident that type Ib synapses were most enriched with abundant mitochondria, with 94% of boutons (22 NMJs, four WT larvae, SD = 11%) housing one or more mitochondria (Fig. 15*B*). type Is synaptic boutons also contained a good amount of strongly-stained mitochondria (71 ± 39% of boutons, 21 NMJs, four larvae; Fig. 15*B*,*C*), although less than type Ib (*p* < 0.05, *t* tests with Bonferroni correction). However, type II synaptic boutons rarely contained visible mitochondrial staining (16 ± 21%, 18 NMJs, four larvae; Fig. 15*C*), which suggests either significantly less mitochondria availability or lower membrane potential to render TMRM staining of existing mitochondria, or both, than type Ib and Is (*p* < 0.001, *t* tests with Bonferroni correction). The lower density of active mitochondria in type II synapses correlates well with its slower Ca^2+^ clearance rate (Fig. 13*B*; Table 3) and higher vulnerability to DNP inhibition (Fig. 14).

## Discussion

Genetically encoded GCaMP indicators are widely used for detecting neuronal circuit activities *in vivo*. However, the analytic power of GCaMP signals has not been fully exploited to extract information regarding basic synaptic physiology. In this study, we took advantage of the special anatomic features of the *Drosophila* larval NMJ to contrast properties of metabotropic aminergic (type II) and ionotropic glutamatergic (tonic type Ib and phasic type Is) synapses using several GCaMP Ca^2+^ indicators. Simultaneous monitoring of GCaMP signals from the three synapses within the same microscopic field demonstrates differential excitability control of Ca^2+^ influx by Na^+^ and K^+^ channels. Analyses of both kinetic and amplitude features of GCaMP signals reveal the extreme effects of particular Na^+^ and K^+^ channels on each of the three synaptic types, as well as the prominent roles of mitochondria-powered Ca^2+^ clearance mechanisms in shaping their distinct Ca^2+^ handling properties.

### The complex nature of GCaMP signals

**Figure 16.**
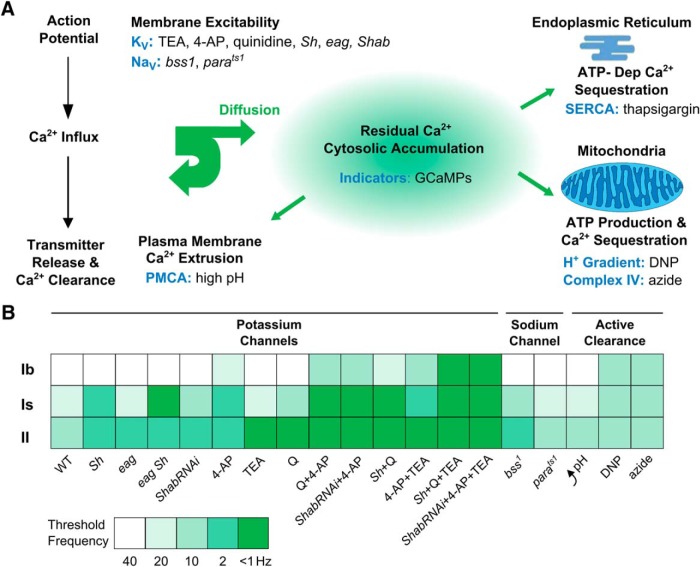
Presynaptic cytosolic residual Ca^2+^ regulation in *Drosophila* NMJ synapses. ***A***, Summary diagram of the relevant cellular mechanisms. The targets of manipulations in this study are shown in blue, including Na^+^ and K^+^ channels (Na_V_ and K_V_), PMCA, and H^+^ gradient and Complex IV of mitochondria, together with the corresponding mutational and pharmacological manipulations. ***B***, A summary diagram comparing effects of specific experimental manipulations on type Ib, Is, and II synaptic terminals of *c164-GCaMP1.3* and *nSyb-GCaMP6m*. The extent of GCaMP signal enhancement is indicated by reduction in the effective frequencies of stimulation. Threshold frequencies for producing clearly detectable GCaMP signals are color coded. Lower threshold frequencies reflect greater excitability or more hampered Ca^2+^ clearance. More than 1 Hz indicates the cases where individual stimuli evoked the giant hallmark GCaMP signals due to extreme hyperexcitability (compare Figs. 5, 6). 4-AP, 200 µM; TEA, 10 mM; and Q, 20 µM quinidine.

We present a summary diagram of how the various genetic and pharmacological manipulations influence Ca^2+^ influx and clearance, hence the amplitude and kinetics of GCaMP signals (Fig. 16). Action potentials, generated and fine-tuned by Na^+^ and K^+^ channels, depolarize synaptic terminals and allows Ca^2+^ influx, which triggers synaptic transmission rapidly in milliseconds (Katz and Miledi, 1967; Jan and Jan, 1976; Wu et al., 1978; Zucker, 1996; Südhof and Rothman, 2009). The influx of Ca^2+^ ions are either actively extruded by PMCA locally (Dipolo and Beaugé, 1979), or sequestered by intracellular organelles such as mitochondria (David et al., 1998) and ER (Klose et al., 2009), or buffered by Ca^2+^ binding proteins (Burnashev and Rozov, 2005). The rise of GCaMP signals spans from hundreds of milliseconds up to seconds before peaking, depending on stimulation frequencies and external Ca^2+^ concentrations (Table 2; Figs. 1–3; cf. Reiff et al., 2005; Tian et al., 2009; Akerboom et al., 2012; Chen et al., 2013). Even with improved sensitivity, GCaMP6 signals are not faster compared to GCaMP1.3, taking at least 100 ms after a single stimulus to reach the peak of fluorescence at high external Ca^2+^ concentration (Chen et al., 2013). Thus, GCaMP signals are several orders slower than individual action potentials and the ensuing postsynaptic potentials (Fig. 9; cf. Xing, 2014; Samigullin et al., 2015). Further, unlike the synthetic Ca^2+^ indicators such as Oregon Green BAPTA (Hill coefficient 1.48; cf. Lnenicka et al., 2006), a GCaMP protein, with calmodulin as the Ca^2+^ sensor, typically binds four Ca^2+^ ions allosterically to produce enhanced fluorescence (Hill coefficients of GCaMP1 = 3.3, GCaMP6m = 2.96; Nakai et al., 2001, Chen et al., 2013). The magnitude of enhancement is thus limited especially at low levels of Ca^2+^ elevations evoked by single action potentials (Rose et al., 2014). Therefore, GCaMP signals better serve as the readout of a leaky integrator that registers cytosolic residual Ca^2+^, i.e., the net Ca^2+^ accumulation as determined by the process of influx and clearance over repetitive firing of action potentials, which can be induced either by trains of stimulation, or hyperexcitability.

In this study, we used low external Ca^2+^ saline to enhance the differentiation power for detecting presynaptic hyperexcitability. Previous electrophysiological studies have shown that hyperexcitable mutations such as *bss*, *eag Sh*, and *Sh;;Shab* cause supernumerary high-frequency firing of action potentials in the NMJ nerve bundle at low Ca^2+^ concentrations (Ganetzky and Wu, 1982a, 1983, 1985; Ueda and Wu, 2006). Therefore, we were able to unequivocally delineate among neighboring synaptic terminals the particular synaptic type that displays hyperexcitability based on GCaMP signals. Simultaneous optical and electrophysiological recordings confirmed that only under extreme hyperexcitability conditions, a single stimulus can rise to a giant GCaMP signal due to high-frequency repetitive firing of action potentials over 100 Hz (Figs. 7–9).

### Distinct K^+^ and Na^+^ channel control of membrane excitability in tonic type Ib, phasic type Is, and aminergic type II synapses

Electrophysiological recording of postsynaptic EJCs or EJPs generally detects the ensemble effects of type Ib, Is, and II synapses. Unlike type Ib and Is synapses, electrophysiological characterization of aminergic type II synapses is more technically challenging because they do not generate readily detectable postsynaptic electrical responses. In contrast, GCaMP signals offer the necessary spatial resolution, and thus enabled demonstration for the first time that mutations or blockers of specific ion channels lead to drastically different effects on type II, as well as type Ib and Is, axonal terminals.

As summarized in Figure 16*B*, our results demonstrated that type Ib synapses were most enriched in the reserve of repolarizing capacity pooled from different K^+^ channel subtypes and could sustain multiple insults of K^+^ channel elimination or blockage before exhibiting the “hallmark” of extreme hyperexcitability (single pulse-evoked giant GCaMP signals at 0.1 mM Ca^2+^; Figs. 5, 6). In comparison, type II synapses had the smallest repertoire of K^+^ channels and simply knocking down either *Shab* or *eag* could induce the hallmark hyperexcitability effect (Fig. 5, right column). In type Is synapses, *Sh* appeared to be the central player for repolarization and perturbing the Sh channel together with either Eag or Shab channels induced the hallmark ceiling effect of extreme hyperexcitability (Fig. 6). This finding also resolved type Is but not Ib motor axons as the major source of the striking electrophysiological phenotype, i.e., axonal high-frequency repetitive firing (Fig. 9; Ganetzky and Wu, 1982a,b, 1985).

Alleles of *para* also have differential effects on type Ib, Is, and II synapses, possibly reflecting differential expression of the Para product, e.g., different splice isoforms (Thackeray and Ganetzky, 1994; Olson et al., 2008; Lin et al., 2012), or posttranslational modifications.

Type II synapses were more prone to conduction failure on high-frequency stimulation, as indicated by GCaMP signals that frequently became intermittent, or even totally missing during 10- to 40-Hz stimulation (Figs. 10, 11). This reflects the well-known axonal passive cable properties; thinner axons have proportionally higher longitudinal internal resistance relative to trans-membrane resistance, resulting in a more limited safety margin of axonal conduction and a longer refractory period for action potentials (Aidley, 1998). Therefore, type II terminals are more prone to K^+^ and Na^+^ channels modifications (Figs. 4–6).

Morphometric analysis confirms that the differential excitability and distinct Ca^2+^ dynamics reported here reflect intrinsic properties of type Ib, Is, and II synapses. The GCaMP responses characteristic of each synaptic type were independent of different sizes of boutons along individual axonal synaptic terminals (Fig. 1; cf. Xing and Wu, 2016), implying that differences in the physical dimensions among the three synaptic bouton types do not contribute to the distinct properties of type Ib, Is, and II synapses reported here.

Obviously, besides Na^+^ and K^+^ channels, other channels may contribute to excitability-controlled Ca^2+^ influx. In particular, different types of Ca^2+^ channels await further study. Notably, previous anatomic studies have shown differences in presynaptic density area among different types of boutons (Atwood et al., 1993; Jia et al., 1993). Ca^2+^ channels are known to be closely associated with active zones embedded within presynaptic density areas. It has been shown that type Is has higher density of active zones than type Ib synapses (He et al., 2009; Lu et al., 2016).

### Acute PMCA suppression and long-term inhibition of mitochondrial ATP production: differential effects on different types of synapses

It should be noted that differences in Ca^2+^ clearance capacity correlate well with the distinct frequency responses in the Ca^2+^ dynamics of these synaptic categories. type II synapses apparently have the slowest rate of Ca^2+^ clearance, as evidenced by its slowest decay of GCaMP signals after secession of stimulation (Figs. 12, 13; Table 3). The faster Ca^2+^ clearance in type Ib than type Is synapses (He et al., 2009) appears to parallel its higher firing frequency (40–60 Hz in Ib vs 10–20 Hz in type Is) during natural bursting activities in semi-intact larval preparations (Cattaert and Birman, 2001; Chouhan et al., 2010), whereas presynaptic cytosolic Ca^2+^ elevation during repetitive firing stimulate mitochondrial oxidative phosphorylation so as to meet temporary burst energy needs (Chouhan et al., 2012). It is conceivable that type Ib synapses thus require a more efficient Ca^2+^ clearance system to avoid intracellular Ca^2+^ build-up. Interestingly, earlier electron microscopy studies have shown that tonic (type Ib) synapses contain more mitochondria than phasic (type Is) synapses in both *Drosophila* larval (Atwood et al., 1993; Jia et al., 1993) and crayfish (Bradacs et al., 1997; Nguyen et al., 1997; Msghina et al., 1998, 1999) NMJs. Our observation using mitochondrial staining confirmed this conclusion and also revealed a far lower density of mitochondria in type II synapses (Fig. 15).

This study showed the importance of mitochondria-powered Ca^2+^ clearance in shaping the distinct dynamics of cytosolic residual Ca^2+^ build-up in type Ib, Is, and II synapses. Inhibiting mitochondrial function with two different means, incubation with either DNP (Fig. 14), a proton ionophore that dissipate mitochondrial proton gradient (Mitchell, 1961; Greenawalt et al., 1964), or azide, an electron-transport chain inhibitor (complex IV), consistently resulted in slower GCaMP signal decay time course and shifted the frequency dependence in type II, Is, and Ib over a period of tens of minutes (Ib, 40–20 Hz; Is, 20–10 Hz).

In contrast to the slow effect of mitochondrial inhibition, high-pH inhibition of PMCA clearly impedes the GCaMP signal decay time course acutely (Figs. 8, 9). Ca^2+^ extrusion via PMCA, a Ca^2+^-ATPase, has been characterized in the *Drosophila* NMJ (Lnenicka et al., 2006; Niggli and Sigel, 2008), as well as goldfish retina (Zenisek and Matthews, 2000). Although under *in vitro* conditions, the fluorescence intensity of GCaMP protein can be affected by pH change (Nakai et al., 2001), intracellularly expressed GCaMP protein is less likely to be affected by extracellular pH manipulation. This notion was supported by lack of change in presynaptic GCaMP baseline fluorescence intensity on external pH changes (*N* = 5 larvae). Therefore, impaired ATP production from mitochondria can lead to PMCA-mediated Ca^2+^ extrusion shut-down (Fig. 14), which could account for the striking effect of long-term DNP incubation.

Notably, DNP treatment significantly impeded the GCaMP signal decay time course only after long-term incubation (beyond 20 min; Fig. 14). Previous studies employing other proton ionophores such as carbonyl cyanide m-chlorophenyl hydrazine (CCCP; Chouhan et al., 2010) has demonstrated that inhibition of mitochondrial proton gradient does not significantly alter overall cytosolic Ca^2+^ dynamics acutely (Lnenicka et al., 2006; Chouhan et al., 2010).

Besides mitochondria, ER may also actively sequestrate Ca^2+^ via sarco/ER Ca^2+^ ATPase (SERCA) in synapses. We inhibited SERCA with thapsigargin (1–2 µM, 1-h treatment) and found no obviously detectable effects on GCaMP signals comparable to the effect of DNP on any of the three types of synapses (in 4 larvae). Previous publications with a higher thapsigargin concentration (10 µM; Klose et al., 2009) or more sensitive Ca^2+^ indicator (Oregon Green BAPTA; Lnenicka et al., 2006) have demonstrated only mildly increased Ca^2+^ signal amplitude and slower time course in type Ib synapses. Therefore, the contributions of mitochondrial and ER Ca^2+^ sequestration may be masked by other high-capacity ATP-dependent Ca^2+^ clearance mechanisms, such as PMCA. Nevertheless, when ATP production by mitochondria is inhibited, these active Ca^2+^ clearance mechanisms could be diminished on gradual depletion of ATP reserve.

In our study, synapses remain viable, and Ca^2+^ clearance system remains functioning for at least tens of minutes, despite mitochondrial inhibition by DNP or azide (note the unimpaired fast Ca^2+^ clearance at 20 min in Fig. 14). Nonmitochondrial sources of ATP such as glycolysis or ATP binding proteins might sustain for some time, until the first sign of depletion, i.e., the appearance of slower GCaMP signal decay kinetics (Fig. 14).

In fact, some vertebrate central nervous system synapses are known to operate without local presynaptic mitochondria (Budd and Nicholls, 1996; Chavan et al., 2015). Similarly, *Drosophila* mutant *drp1* and *dMiro* larval NMJs, with greatly reduced numbers of synaptic mitochondria, remain viable and display essentially normal Ca^2+^ dynamics and buffer capacity unless challenged by prolonged stimulation beyond minutes (Guo et al., 2005; Verstreken et al., 2005; Chouhan et al., 2010). In our studies, type II synapses had a lower abundance in mitochondria and thus more limited ATP reserve and in consequence were most vulnerable to DNP treatment. They were the first to show lengthened decay and to become completely nonresponsive subsequently during DNP incubation (Fig. 14).

### Interpretation of synaptic GCaMP signals

Overall, our study indicates that analysis of GCaMP signals can be extended to extract information about specific synaptic physiologic properties. GCaMP signals offer higher spatial resolution and can complement electrophysiology data to pinpoint critical differences in channel expression and excitability properties among neighboring synaptic terminals.

Systematic kinetic analysis of GCaMP signals revealed the predominant effects of hyperexcitability on the rise kinetics and Ca^2+^ clearance capacity on the decay kinetics. In conjunction with focal electrophysiological recording, genetic and pharmacological analyses indicate a close relationship between GCaMP signals and cytosolic residual Ca^2+^ accumulation rather than the rapid process of Ca^2+^ influx that triggers transmitter release. This approach also revealed the striking hyperexcitable effects caused by insults to multiple K^+^ channels, leading to the hallmark giant GCaMP signals evoked by single stimuli that generated high-frequency supernumerary firing of nerve action potentials. Thus, GCaMP signals may be further exploited to shed new light on activity-dependent plasticity in synapses of distinct properties. This work may help to establish guidelines for refined interpretations of GCaMP signals beyond the first-order, qualitative indications for gross neuronal activities in neural circuits.

## Synthesis

Reviewing Editor: Miriam Goodman, mbgoodman@stanford.edu Decisions are customarily a result of the Reviewing Editor and the peer reviewers coming together and discussing their recommendations until a consensus is reached. When revisions are invited, a fact-based synthesis statement explaining their decision and outlining what is needed to prepare a revision will be listed below. The following reviewer(s) agreed to reveal their identity: Gaiti Hasan. Note: If this manuscript was transferred from JNeurosci and a decision was made to accept the manuscript without peer review, a brief statement to this effect will instead be what is listed below.

This manuscript is a remarkably comprehensive study describing the synaptic properties of three different classes of synaptic boutons at the Drosophila NMJ, as visualized by GCaMP imaging. The primary aim is to delineate the nature of membrane channels and Ca2+ homeostatic mechanisms that shape GCaMP signals in the three classes of synapses studied. The authors systematically stimulate Type 1b, Type1s and Type II synapses at various frequencies and quantify the GCaMP responses obtained in each case.

The authors show that each synaptic class has a different stimulation threshold that is related to the external Ca2+ concentration in a synapse-specific manner. They then go onto identify membrane channels (K+ and Na+) that regulate these Ca2+ responses. For these studies, they have used both mutants and pharmacological reagents to investigate the contribution of voltage-gated ion channels and of mechanisms of calcium clearance for shaping GCaMP signals. This last analysis is based solely on pharmacological studies. The manuscript will serve as an excellent starting point for many investigators in helping understand how GCaMP signals vary among presynaptic terminals and provide guidance for interpreting signal dynamics.

The two reviewers had suggestions to improve the manuscript in revision and recommended the manuscript for publication in eNeuro. Please note that concerns raised by Reviewer 1 regarding the contribution of the ER calcium pump, SERCA, can be addressed by discussing this additional factor in the text of manuscript. Additional experimental work is not required. If, on the other hand, such experiments have already been performed, their inclusion in this study would increase its breadth. Similarly, Reviewer 2 raised a concern regarding possible artifacts of using DNP to manipulate the contribution of mitochondria to GCaMP signal dynamics and suggested directly observing mitochondrial calcium dynamics. In this regard, it would be sufficient to include additional discussion regarding the role of mitochondria.

Please find the reviewers specific suggestions below

Reviewer 1

1) Re-organize Figs 4 and 5 as one figure. This would help the reader understand the double mutant phenotype in the context of the phenotype shown by the single mutant.

2) In the text and figure legend, please explain "Q" before the results for Figs 6 and 7.

3) The data for the conclusion in lines 377-379 is described in the subsequent paragraphs. Perhaps this concluding sentence could be moved to after describing data for Figs 6 and 7?

4) Paragraph starting from lines 408- 414 needs to be revised. Type 1b synapses do not show a response at 2Hz stimulation under any of the conditions shown (Fig 7A, left column) as mentioned in lines 411-412. The results obtained in Fig 7 need to be described in greater detail.

5) Line 543 - delete the word "in".

6) Neurons also express the ER- Ca2+ pump SERCA for which there exist established pharmacological inhibitors as well as mutants. The authors should discuss the possibility that synapses may differ in their ability to clear cytosolic calcium based on SERCA expression and function, and this can be tested with existing reagents. In Figure 17 they should mention SERCA at the top right where they show ATP-dep Ca2+ clearance by ER.

7) Check line 1191 - there appears to be some words missing in the sentence.

Reviewer 2

1) Taking the information from the statistics table at line 248 on page 11 of the manuscript, there are many figures that have normality, and others where they do not, and for the ones that do not, the authors correctly use non-parametric statistics such as KW. When I follow this then to the actual figure, the authors appear to show graphs that have mean and SD or standard deviation. For non parametric tests, this is not informative, and I question whether the correct visual graph is used in these circumstances. Can the authors please address this or confirm why such graphs are used to represent non-parametric data throughout the manuscript?

2) Data derived for figure 14 is dependent on the use of high pH to knock out the function of the PMCA. One concern I have is how a pH of 8.8 effects the actual fluorophore GCaMP since such fluorophores are very sensitive to pH? Can this be addressed to determine if the observed result is not a result of pH directly on the GCaMP? Also, has these findings about the PMCA already been established?

3) The use of DNP to investigate Ca2+ dynamics leads to some concern since it has been shown to significantly depolarize the cell membrane causing increased Ca2+ conductance from the voltage gates Ca2+ channels. Is there a way to directly observe mitochondrial Ca2+ dynamics? I strongly believe the authors should state this caveat in their observations. Between paragraphs of line 632 page 31 and 633, this would be a good transition for this line of reasoning and justify the use of azide.
