# Erratum: Xing and Wu, “Unraveling Synaptic GCaMP Signals: Differential Excitability and Clearance Mechanisms Underlying Distinct Ca2+ Dynamics in Tonic and Phasic Excitatory, and Aminergic Modulatory Motor Terminals in *Drosophila*.”

**DOI:** 10.1523/ENEURO.0096-21.2021

**Published:** 2021-04-26

In the article, “Unraveling Synaptic GCaMP Signals: Differential Excitability and Clearance Mechanisms Underlying Distinct Ca2+ Dynamics in Tonic and Phasic Excitatory, and Aminergic Modulatory Motor Terminals in *Drosophila*,” by Xiaomin Xing and Chun-Fang Wu, which published online on February 8, 2018, a fly stock genotype appeared incorrectly because of an author error. In the fourth paragraph of the Fly stocks subsection of the Materials and Methods, “*y1 v1; P{TRiP.JF02146}attP2*” should be replaced with “y^1^ sc* v^1^ sev^21^; P{TRiP.HMS02400}attP40.” The online version has been corrected.

